# In vitro and in vivo efficacy of thiacloprid against *Echinococcus multilocularis*

**DOI:** 10.1186/s13071-021-04952-7

**Published:** 2021-09-06

**Authors:** Chuanchuan Liu, Haining Fan, Jie Ma, Lan Ma, Ri-li Ge

**Affiliations:** 1 Research Center for High Altitude Medicine, Qinghai University, Xining, 810001 Qinghai China; 2 Qinghai University Affiliated Hospital, Xining, 810001 Qinghai China; 3 Qinghai Key Laboratory for Echinococcosis, Xining, 810001 Qinghai China; 4 Qinghai Key Laboratory of Science and Technology for High Altitude Medicine, Xining, 810001 Qinghai China; 5 Qinghai-Utah Joint Research Key Lab for High Altitude Medicine, Xining, 810001 Qinghai China

**Keywords:** *Echinococcus multilocularis*, Thiacloprid, Neonicotinoids, Lymphocytes, Cytokines, Matrix metalloproteinases

## Abstract

**Background:**

Alveolar echinococcosis (AE) is a chronic zoonosis caused by the larval form of *Echinococcus multilocularis *(*E. multilocularis*). Current chemotherapy against AE has relied on albendazole and mebendazole, which only exhibit parasitostatic and not parasiticidal efficacy. Therefore, novel compounds for the treatment of this disease are needed.

**Methods:**

Phosphoglucose isomerase (PGI) assays were used for compound screening of seven neonicotinoids. The anti-parasitic effects of thiacloprid were then evaluated on *E. multilocularis* metacestode vesicles, germinal cells and protoscoleces in vitro. Human foreskin fibroblasts (HFF) and Reuber rat hepatoma (RH) cells were used to assess cytotoxicity. Glucose consumption in *E. multilocularis* protoscoleces and germinal cells was assessed by measuring uptake of 2-deoxyglucose (2-DG). Molecular docking was used to evaluate the potential binding sites of thiacloprid to acetylcholine receptors. In vivo efficacy of thiacloprid was evaluated in mice by secondary infection with *E. multilocularis*. In addition, ELISA and flow cytometry were used to evaluate the effects of cytokines and T lymphocyte subsets after thiacloprid treatment. Furthermore, collagen deposition and degradation in the host lesion microenvironment were evaluated.

**Results:**

We found that thiacloprid is the most promising compound, with an IC_50_ of 4.54 ± 1.10 μM and 2.89 ± 0.34 μM, respectively, against in vitro-cultured *E. multilocularis* metacestodes and germinal cells. Thiacloprid was less toxic for HFF and RH mammalian cell lines than for metacestodes. In addition, thiacloprid inhibited the acetylcholinesterase activity in protoscoleces, metacestodes and germinal cells. Thiacloprid inhibited glucose consumption by protoscoleces and germinal cells. Subsequently, transmission electron microscopy revealed that treatment with thiacloprid damaged the germinal layer. In vivo, metacestode weight was significantly reduced following oral administration of thiacloprid at 15 and 30 mg/kg. The level of CD4^+^ T lymphocytes in metacestodes and spleen increased after thiacloprid treatment. Anti-echinococcosis-related cytokines (IL-2, IL-4, IL-10) were significantly increased. Furthermore, thiacloprid inhibited the expression of matrix metalloproteinases (MMPs 1, 3, 9, 13) and promoted collagen deposition in the host lesion microenvironment.

**Conclusions:**

The results demonstrated that thiacloprid had parasiticidal activity against *E. multilocularis *in vitro and in vivo, and could be used as a novel lead compound for the treatment of AE.

**Graphical abstract:**

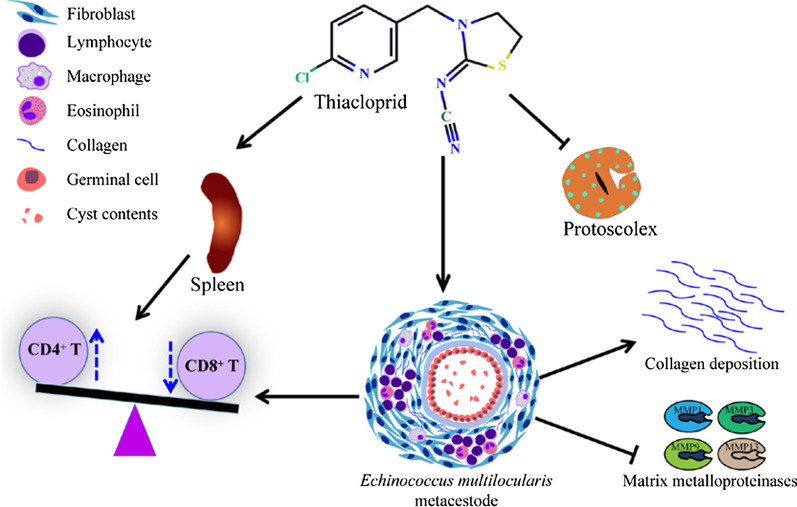

**Supplementary Information:**

The online version contains supplementary material available at 10.1186/s13071-021-04952-7.

## Background

Alveolar echinococcosis (AE) is a rare zoonotic parasitic disease caused by the metacestode stage of *Echinococcus multilocularis*, which presents unclearly delimited foci (alveococcus) located in the liver (in 99% of cases) [1]. The exogenous gemmation of AE can penetrate into the surrounding tissue; it presents tumor-like features and can form metastases in distant organs through the blood and lymphatic system. Incidence of *E. multilocularis* has so far been restricted to the Northern Hemisphere [1]. The life cycle of echinococcus involves two mammalian hosts, usually including a definitive host of canids or foxes and an intermediate host of small rodents [2]. Humans are aberrant hosts and become infected by orally ingesting *E. multilocularis* eggs from the environment. Oncospheres pass through the portal and reach the liver, where they usually settle and develop as larvae (metacestodes) [3]. The interaction between the parasite and the host leads to the formation of infectious granuloma reaction [4–6].

The ideal treatment for AE is still radical surgery, accompanied by chemotherapy. However, surgery is reserved for early-stage disease when lesions can be completely resected with a safe margin (≥ 2 cm) of unaffected tissue and no distant metastases [7]. Because most cases are found at an advanced stage, only 35% of patients can undergo curative surgery [7]. In inoperable cases, chemotherapy alone is applied. Systemic anti-infective treatment relies on continuous administration of two benzimidazole carbamates, albendazole (ABZ) and mebendazole (MBZ), which are the only anti-infective drugs clinically effective in interrupting larval growth of *Echinococcus* spp*.* [8, 9]. Stem cells of *E. multilocularis* are more resistant to benzimidazoles than other cells of the parasite, and this, together with the limited uptake and half-life of benzimidazoles, leads to a parasitostatic rather than parasiticidal effect [10]. In addition, benzimidazoles are not always well tolerated and can cause serious side effects such as hepatotoxicity in some patients [11]. Thus, improved drug treatments are urgently needed.

Drugs currently on the market, or under development for other indications, are being explored to identify novel treatment options for AE. Repurposed drugs mainly include broad-spectrum anti-infective drugs, drugs that inhibit cellular proliferation (such as anti-cancer compounds), natural products, and substances that are active against other pathogens [12, 13]. Neonicotinoids are important insecticides that have been developed over the last three decades (Additional file 1: Figure S1). Neonicotinoids are recognized as systemic insecticides used to protect crops from penetrating insects and to control the body surface parasites of cats and dogs [14]. The insecticidal activity of neonicotinoids occurs largely through their excitatory effect on the nicotinic acetylcholine receptors (nAchRs) of the insect’s postsynaptic membrane, thereby causing acetylcholine to accumulate and leading to insect paralysis and death [15]. Neonicotinoids are also effective acetylcholinesterase inhibitors [16]. Furthermore, their low affinity for several subsets of mammalian nAChRs in binding assays suggests that neonicotinoids are relatively safe for mammals including humans [17]. Unlike nicotine, neonicotinoids have low toxicity and pose little risk to mammals [18]. They cannot easily penetrate the blood–brain barrier [19] and fail to accumulate in animal tissues [20]. As reported by Schwabe et al., hydatid scoleces and brood capsules were found to contain special acetylcholinesterase [21]. Koziol et al. highlighted the lack of a cholinergic component in the protoscolex and cyst wall of *E. multilocularis* [22]*.* Anthelmintic drugs that are currently marketed act mainly on nematode nAchRs [23], but are ineffective against echinococcus.

The neonicotinoids against *E. multilocularis* metacestode vesicles were screened in vitro, and thiacloprid showed the effect against metacestodes (Additional file 2: Figure S2). In this study, we evaluated the in vitro anthelmintic effects of thiacloprid on metacestodes, germinal cells and protoscoleces of *E. multilocularis*. We further investigated the in vivo efficacy and cytotoxicity of thiacloprid in experimentally infected mice.

## Methods

### Animals and ethics statement

Specific pathogen-free (SPF) BALB/c mice (female, 18–20 g) and Mongolian gerbils (male, 60–80 g) were supplied by the Nanjing Qinglongshan animal breeding base. Animals were housed in temperature-controlled (23 ± 1 °C), light-cycled (12 h light/dark cycle) individual ventilated cages. Food and water were provided ad libitum. All the operations were conducted under 0.3% pentobarbital sodium anesthesia, and all efforts were made to minimize the pain of animals. All animal experiments were performed in accordance with the regulations of the Ministry of Science and Technology of China and the administrative measures of experimental animals in Qinghai Province, which were approved by the Institutional Review Board of the Medical College of Qinghai University (IACUC-201905010005) and the Qinghai University Affiliated Hospital (AF-RHEC-0018-01).

### Cells and chemicals

Human foreskin fibroblasts (HFF) and Reuber rat hepatoma (RH) cells were purchased from Procell (Wuhan, China). Dulbecco’s modified Eagle medium (DMEM), Medium 199 and fetal bovine serum (FBS) were obtained from Gibco (Auckland, New Zealand). Solutions containing trypsin–EDTA, penicillin-streptomycin (100×) and gentamicin were purchased from Procell. All chemicals were purchased from Sigma-Aldrich (St. Louis, MO, USA), unless stated otherwise. All neonicotinoids were prepared as 10 mM stock solutions in dimethyl sulfoxide (DMSO) upon arrival and stored at –20 °C.

### *E. multilocularis* metacestode in vitro cultivation

Larval material (isolate Qinghai) was obtained from female BALB/c mice experimentally infected with *E. multilocularis* homogenized larval tissue, which was originally isolated from a naturally infected plateau pika (*Ochotona curzoniae*) collected in Yushu, Qinghai province, China. Molecular identification of the isolate of *E. multilocularis* is described by Li et al. [24]. *Echinococcus multilocularis* metacestodes were prepared and cultured as described previously [25]. Briefly, *E. multilocularis* metacestode tissue material was retrieved from euthanized mice that had been intraperitoneally infected with *E. multilocularis* metacestodes for 3 months. The isolated metacestodes were crushed through a metal tea strainer, and incubated overnight in phosphate-buffered saline (PBS) containing 1% penicillin/streptomycin. Then, 1 ml metacestode tissue was co-cultured with 5 × 10^6^ Reuber rat hepatoma (RH) feeder cells in DMEM containing 10% FBS and 1% penicillin/streptomycin at 37 °C and 5% CO_2_, with medium changes once a week. In vitro-cultured metacestode vesicles were used for experiments when they reached diameters of 2–4 mm [26]. Some of the vesicles were fixed with 4% paraformaldehyde for pathological examination.

### Confirmation of metacestode vesicles by RT-PCR

Metacestode vesicles (without host cell contamination) were detected by reverse transcription polymerase chain reaction (RT-PCR). Total RNA was extracted from metacestode vesicles and host tissue (normal mouse liver) using the RNAsimple Total RNA Kit (TianGen Biotech, Beijing, China) according to the manufacturer’s instructions. The first-strand cDNA was synthesized using the FastKing gDNA Dispelling RT SuperMix Kit (TianGen Biotech). PCR was performed using primers for the specific amplification of *E. multilocularis* GAPDH [27] and mouse GADPH (forward: 5′-CGTGGGGCAGCCCAGAACAT-3′; reverse: 5′-GAGCAATGCCAGCCCCAGCA-3′).

The RT-PCR reaction was performed using the Mastercycler Nexus PCR apparatus in a final volume of 50 μl: 25 μl 2× Taq PCR Mastermix (TianGen Biotech), 1.2 μl of each primer (10 μM), 2 μl cDNA product, 20.6 μl ddH_2_O. Amplification was performed using the following conditions: 3 min at 94 °C followed by 30 cycles of 94 °C for 30 s, 60 °C for 30 s and 72 °C for 20 s, and a final extension step at 72 °C for 3 min. PCR products were separated by 2% agarose gel electrophoresis and stained with GeneRed (TianGen Biotech) for visualization under UV light. Metacestode germinal cells were subsequently identified in the same manner.

### In vitro assessment of thiacloprid against *E. multilocularis* metacestodes

The efficacy of thiacloprid against the *E. multilocularis* metacestode was evaluated by applying a phosphoglucose isomerase (PGI) assay that measures the release of the enzyme phosphoglucose isomerase upon physical impairment of metacestodes [26]. In brief, medium without phenol red (DMEM, 1% penicillin/streptomycin sulfate, 2 mM l-glutamine) was added to the same volume of vesicles and distributed to 48-well (12–15 per vesicles) plates. Subsequently, metacestodes were incubated for 5 days with different concentrations (0, 0.06, 1.2, 2.5, 5, 10, 20, 40, 80 and 160 μM) of thiacloprid or praziquantel (PZQ), after which PGI release was quantified exactly as described in Stadelmann et al. [28]. Triton X-100 (0.1% in PBS) was applied as a positive control (maximal release of vesicle fluid). Each condition was tested in biological triplicate. After 5 days of incubation, 200 μl medium supernatant was collected from each well and stored at − 20 °C until further measurements were performed. PGI measurements were performed as described earlier [26], except that an Infinite M200 PRO reader (Tecan, Männedorf, Switzerland) was used to measure the increase in absorbance at 340 nm. PGI activity was calculated using the EC_50_ calculator (https://www.aatbio.com/tools/ec50-calculator) from the linear regression of the enzyme reaction over time and presented as a percentage relative to the values obtained by treatment of vesicles with 0.1% Triton X-100.

### Assessment of in vitro toxicity in human foreskin fibroblasts and Reuber rat hepatoma cells

An alamarBlue assay was used to assess the toxicity of thiacloprid to confluent and pre-confluent mammalian cells in vitro [29]. Human foreskin fibroblasts (HFF, Procell) and rat hepatoma cells (RH, Procell) were seeded into 96-well cell culture plates in DMEM supplemented with 10% FBS and 1% penicillin–streptomycin at 37 °C and 5% CO_2_. For detecting the growth inhibitory effects of confluent cells, HFF and RH cells were seeded at 10,000 cells per well and 50,000 cells per well, respectively. After overnight culture, thiacloprid or PZQ was added and diluted in serial concentrations (0, 0.6, 1.2, 2.5, 5, 10, 20, 40, 80 and 160 μM). For detecting growth inhibitory effects on proliferating cells, cells were seeded at 1000 (HFF) and 5000 (RH) cells per well. Thiacloprid was added after 5 h of cell attachment. To measure the viability of the cells after treatment for 5 days, the alamarBlue assay was employed and viability calculated as described previously [29].

### Assessment of in vitro toxicity in *E. multilocularis* germinal cells

To evaluate the activity of thiacloprid against parasitic stem cells, germinal cells from in vitro metacestode vesicles were investigated as described by Spiliotis et al. [30]. Briefly, 20 units of cells were distributed into a black 384-well plate. Different concentrations of thiacloprid or PZQ (0, 0.06, 1.2, 2.5, 5, 10, 20, 40, 80 and 160 μM) were added to the cells. After culture at 37 °C for 5 days under a humid nitrogen atmosphere, 25 μl CellTiter-Glo containing 1% Triton X-100 was added. The plates were incubated at room temperature in the dark for 15 min. After the total destruction of the cellular aggregates, luminescence was measured using an Infinite M200 PRO reader (Tecan), and 0 μM values were set to 100% viability. The IC_50_ values were calculated using an online IC_50_ calculator after logit-log transformation. Four independent replicates were conducted.

### Preparation of *E. multilocularis* protoscoleces

The protoscoleces were isolated from metacestodes in Mongolian gerbils (Additional file 3: Figure S3). Metacestodes were removed aseptically from the abdominal cavities of Mongolian gerbils in a biosafety cabinet after euthanasia. The metacestodes were sliced in 1× PBS and filtered through four layers of sterile gauze into a 50-ml sterile centrifuge tube. The protoscoleces were first filtered by a 100-μm cell strainer, and then the calcified bodies were removed with a 40-μm cell strainer. The protoscoleces naturally settled and were washed with PBS 8–10 times as backup.

### Efficacy of thiacloprid against *E. multilocularis* protoscoleces in vitro

Viable protoscoleces were cultured in Medium 199, supplemented with 1% penicillin–streptomycin, 50 μg/ml gentamicin and 4 mg/ml glucose according to a previously described method [31]. Protoscoleces were added into six-well cell culture plates and incubated for 5 days with thiacloprid (0, 0.06, 1.2, 2.5, 5, 10, 20, 40 and 80 μM). PZQ was used as a positive control. The protoscoleces were cultured in an incubator at 37 °C and 5% CO_2_ for 7 days. Additionally, 0.1% eosin exclusion test was used to evaluate the viability of protoscoleces. To reduce the bias as much as possible, protoscolex viability was observed by two experimenters under double-blind conditions. Each experiment was conducted three times.

### Determination of cholinesterase activity in *E. multilocularis* protoscoleces, metacestodes and germinal cells

The effects of thiacloprid on the acetylcholinesterase activity of *E. multilocularis* protoscoleces, metacestodes and germinal cells were determined according to a previously described method [32, 33]. The protoscoleces, metacestode and germinal cells were washed twice with PBS and homogenized with an electric homogenizer in precooled PBS. Centrifugation of the homogenates was performed at 100,000×*g* for 30 min. The protein concentration of the supernatant was determined by the modified Lowry method according to the instructions, and the concentration was adjusted to 2 mg/ml. Thiacloprid (5 μM) was added to the extract and incubated at 37 °C for 10 min. The extract was supplemented with 0.1% DMSO as control. Subsequently, acetylcholinesterase activity was assessed according to Ellman’s procedure [33] using a microplate with 260 µl of 0.1 M PBS buffer pH 8.0, 10 µl of 10 mM dithiobisnitrobenzoic acid solution, 2.5 µl of 75 mM substrate (acetylthiocholine iodide) and 25 µl of the sample treated with thiacloprid. The OD increment was measured at 412 nm using an Infinite M200 PRO reader (Tecan). Each experiment was conducted three times. BW284c51 (CAS number: 402-40-4, Sigma) was used as a positive control.

### Glucose consumption

Glucose consumption in *E. multilocularis* protoscoleces and germinal cells was detected using the Screen Quest Colorimetric Glucose Uptake Assay Kit (AAT Bioquest, Sunnyvale, CA, USA). The newly separated protoscoleces were washed twice with Krebs–ringer phosphate HEPES (KRPH) buffer and then resuspended in 1 ml glucose uptake buffer for 1 h. Each well contained about 200 protoscoleces. Isolated *E. multilocularis* germinal cells were resuspended with serum-free DMEM and cultured overnight in nitrogen at 37 °C. Germinal cells were then washed twice with KPRH buffer and resuspended in 90 μl glucose uptake buffer for 1 h. Subsequently, the protoscoleces and germinal cells were added with 10 μl thiacloprid (5 μM) for 1 h, and treated with 10 μl 2-deoxyglucose (2DG) for 1 h. The germinating cells treated with thiacloprid were labeled with mitochondria red fluorescent probe (Beyotime, Shanghai, China) and the percentage of living cells was measured by flow cytometry. The protoscoleces and germinal cells were washed twice with KRPH buffer and then lysed with 25 μl acidic lysis buffer in an Eppendorf tube (EP) for 30 min. A neutralizing buffer was added to each well and left at room temperature for 20 min to neutralize the lysate. Fifty microliters of 2DG uptake assay working solution was added to each tube and incubated at room temperature for 2 h. Lastly, the mixture was transferred to a 96-well cell culture plate and the absorbance was measured at 570 nm using an Infinite M200 PRO reader (Tecan). Albendazole sulfoxide (ABZSO, 15 μM) was used as a positive control [34]. Results are presented as a percentage relative to the values obtained without treatment.

### Toxicity of thiacloprid in vivo

Based on the previous thiacloprid dose concentration, we selected 15 mg/kg and 30 mg/kg thiacloprid concentrations to evaluate the toxicity in vivo [35]. The toxicity of thiacloprid was assessed in normal BALB/c mice. Normal mice were randomly divided into three groups and received the following treatments (6 animals per group): The control group was not administered any drug; the Thia15 group and Thia30 group received thiacloprid orally (15 mg/kg/day and 30 mg/kg/day). After 6 weeks of treatment, blood samples were collected from the orbital sinus of mice under anesthesia before euthanasia, which were preserved in EDTA-K2 anticoagulant tubes for white blood cell (WBC), hemoglobin (Hb) and platelet (PLT) analysis. For assessing biochemical indicators, blood samples were incubated in anticoagulant-free tubes at 37 °C for 1 h, followed by 3500 rpm centrifugation for 10 min. The isolated serum samples were used to detect relative levels of alanine aminotransferase (ALT), aspartate aminotransferase (AST), total bilirubin (TBIL), direct bilirubin (DBIL), indirect bilirubin (IBIL), total protein (TP), albumin (ALB), alkaline phosphatase (ALP), creatinine (CREA) and blood urea nitrogen (BUN). Subsequently, mice were sacrificed for harvesting the liver and kidneys, which were fixed in 4% paraformaldehyde, prepared for hematoxylin–eosin (HE) staining and observed under a Zeiss Vert.A1 microscope.

### In vivo effect of thiacloprid treatment in mice experimentally infected with *E. multilocularis* metacestodes

In vitro-grown metacestode vesicles were pressed through a 500 µm mesh and washed three times with sterile PBS. The parasite tissue was then taken up in an equal volume of sterile PBS. Each mouse was intraperitoneally injected with 200 μl suspension. Seven mice were injected with 200 μl PBS as the control group. Twenty-eight infected mice were randomly distributed into four groups (7 mice/group): the untreated group was not administered any drug; the ABZ group was treated with ABZ orally (100 mg/kg/day); and the Thia15 group and Thia30 group received thiacloprid orally (15 mg/kg/day and 30 mg/kg/day). Treatments of mice started 2 weeks post-infection, and treatments were administered daily for 6 weeks. After 6 weeks of treatment, whole blood via the orbital sinus was collected from mice and the animals were euthanized by CO_2_. Blood samples were incubated at 37 °C for 1 h and centrifuged at 3500 rpm for 10 min at 4 °C. The serum was harvested and stored at –20 °C for IL-2, IL-4, IL-10 and IgE detection. All metacestode tissues were collected, and the total wet weight of metacestodes per mice was determined. Furthermore, the content of cytokines in microcyst fluid was detected by enzyme-linked immunosorbent assay (ELISA). Several metacestode tissue specimens were processed for transmission electron microscopy (TEM) and histopathological experiments.

### Hematoxylin–eosin (HE) staining

Tissues from experimental animals were washed in PBS and fixed in 4% paraformaldehyde at room temperature for 36 h. On the next day, they were dehydrated, embedded, and sliced into 5-μm sections. After dewaxing and dehydrating, sections were stained with hematoxylin for 2–5 min, and re-stained with eosin for 15 s. They were sealed with neutral gum and observed under a BX51 microscope (Olympus, Tokyo, Japan).

### Periodic acid Schiff (PAS) staining

A Periodic Acid Schiff Stain Kit (Solarbio, Beijing, China) was used to show the PAS-positive laminated layer characteristic of *E. multilocularis* metacestodes. The section (5 μm) was dewaxed in xylene and rehydrated in 100%, 95%, 80%, and 75% alcohol baths. Staining was then carried out according to the kit instructions.

### Sirius red staining analysis

The metacestodes were fixed with 4% paraformaldehyde and embedded in paraffin to make sections with a thickness of 5 μM. The sections were dewaxed with water, stained with Picrosirius red for 10 min, rinsed and dehydrated with anhydrous ethanol, and sealed with neutral balsam after the xylene was transparent. Each section of 400× visual field image was randomly collected. The ratio of the area of all images collected to the area of the visual field was measured using the ImageJ analysis system, and the percentage of collagen fibre was calculated.

### T lymphocyte subset analysis

The spleens of mice were removed and then weighed to calculate the spleen index. The spleen tissue was cut, softly pressed and dispersed by passing through 70-μm cell strainers. Metacestode tissues were cut into pieces and then digested by 0.2% collagenase II to generate a single-cell suspension. The lymphocytes were separated based on gradient centrifugation with a lymphocyte separation kit (Tbdscience, Tianjin, China). The lymphocytes were incubated with FITC-conjugated anti-CD3 (BioLegend, San Diego, CA, USA), PE-conjugated anti-CD4 (BioLegend), and APC-conjugated anti-CD8 (BioLegend) antibodies, and then analyzed by flow cytometry using a NovoCyte flow cytometer (ACEA/Agilent, Santa Clara, CA, USA).

### Enzyme-linked immunosorbent assay (ELISA)

ELISA was employed to detect serum IL-2, IL-4, IL-10 and IgE following the manufacturer's instructions. Mouse collagen I and collagen III ELISA kits were used to measure the levels of collagen I and collagen III in the cell culture supernatant after intervention with thiacloprid. All ELISA kits were provided by Elabscience Biotechnology Co., Ltd., Wuhan, China. The OD value at 450 nm was read with a Tecan Infinite M200 PRO enzyme reader (Männedorf, Switzerland), and the concentration was calculated according to the standard curve.

### Scanning electron microscopy (SEM) and transmission electron microscopy (TEM)

The variations in the microstructure of the protoscoleces or metacestodes were observed under an electron microscope [28]. In vitro-cultured *E. multilocularis* protoscoleces and metacestodes were treated with thiacloprid at 0 and 5 μM for 5 days at 37 °C and 5% CO_2_. They were washed twice in PBS and then fixed for 3 h with 2.5% glutaraldehyde (pH = 7.2); next, they underwent 2 h post-fixation in 2% OsO_4_ (SPI-CHEM, West Chester, PA, USA). Subsequently, the samples were washed in double-distilled water and treated with 1% uranyl acetate for 25 min. After the samples were washed again in double-distilled water, dehydration was conducted in ethanol at continuous gradients (30–50–70–80–90–95–100%) over a period of 10 min. For SEM analysis, the dehydrated samples were dipped into hexamethyldisiloxane and then air-dried in a fume hood. The sample was observed under a Hitachi SU8100 scanning electron microscope (Tokyo, Japan) after gold spraying. For TEM analysis, the dehydrated samples passed through a dehydrating agent, and an epoxy resin (SPI-CHEM) permeated and polymerized at 60 °C overnight. A 50-nm ultra-thin section was prepared and then loaded on a 300-mesh copper grid. It was stained with uranyl acetate and lead citrate (SPI-CHEM). The sample was observed under a Hitachi HT7700 transmission electron microscope. To reduce the bias, electron microscopy was observed by professionals in the case of an unknown group.

### Real-time qPCR

Total RNA was extracted with TRIzol Reagent (Thermo Fisher Scientific, Waltham, MA, USA) according to the manufacturer’s instructions. The purity and concentrations of RNA were quantified using NanoDrop 2000 absorbance measurements at 260/280 nm. Total RNA was converted into cDNA sequences using the FastKing gDNA Dispelling RT SuperMix Kit (TIANGEN Biotech), and RT-qPCR was performed using the RealUniversal Color PreMix Kit (TIANGEN Biotech) on an ABI QuantStudio 5 system (Applied Biosystems, Waltham, MA, USA). The specific primers are shown in Additional file 4: Table S1. The PCR cycling conditions were as follows: pre-denaturation at 95 °C for 15 min followed by 40 cycles at 95 °C for 10 s and 60 °C for 34 s. The relative mRNA expression levels were quantified using the 2^−ΔΔCT^ method. RPS18 served as an internal control.

### Western blotting analysis

A total protein extraction kit (Solarbio) was used to extract cell or tissue total protein. The protein concentration was determined by the protein quantitative assay kit (Thermo Fisher Scientific). Then, electrophoresis was performed with 20–50 μg of protein sample. The protein was separated by sodium dodecyl sulfate polyacrylamide gel electrophoresis (SDS-PAGE) and transferred to a 0.22 μm PVDF membrane (Merck Millipore, Darmstadt, Germany). The PVDF membrane was blocked with 5% of nonfat powdered milk in Tris-buffered saline Tween 20 (TBST) for 1 h at room temperature. The membrane incubation with primary antibodies was performed at 4 °C overnight. The antibody information is listed in Additional file 5: Table S2. The membrane was washed with TBST and incubated with HRP-labeled secondary antibodies (1:5000, Abclonal, Wuhan, China) for 1 h at room temperature. Blotted membranes were visualized using enhanced chemiluminescence reagents (Thermo Fisher Scientific). Protein expression was depicted as the intensity of each protein relative to that of β-actin using ImageJ software.

### Statistical analysis

The data are presented as the mean ± standard deviation (SD) and were plotted with GraphPad Prism 8.0 software. The data for both groups were analyzed using unpaired two-samples *t*-tests. Multiple comparisons between more than two groups were analyzed using by one-way analysis of variance (ANOVA) or Kruskal–Wallis test (non-parametric), and IC_50_ and EC_50_ values were calculated using an online half-max graphing calculator (https://www.aatbio.com/index.html). Values of *P* < 0.05 were considered statistically significant.

## Results

### In vitro activity of thiacloprid against *E. multilocularis* metacestodes

**Fig. 1:**
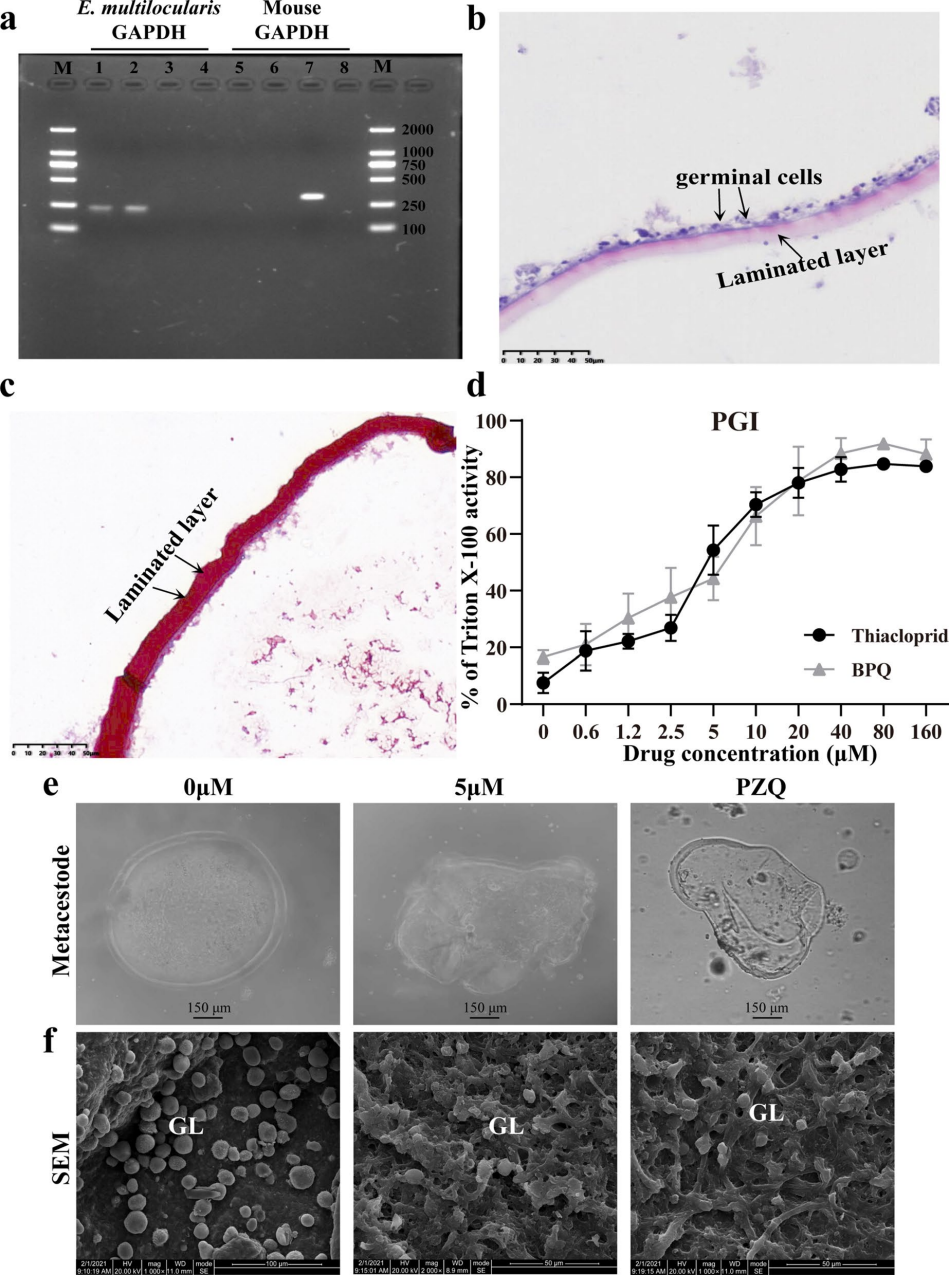
Thiacloprid against *E. multilocularis* metacestodes. **a** Identification of metacestode and germinal cell. *Echinococcus multilocularis* GAPDH and mice GAPDH were used for the characterization of *E. multilocularis* metacestodes and germinal cells (without host cell contamination). Lanes 1 and 5: metacestode; lanes 2 and 6: germinal cells; lanes 3 and 7: mouse liver; lanes 4 and 8: negative control. **b** Pathological observation of metacestode. Obvious germinal layer and laminated layer were observed in the metacestode section after HE staining. Scale bar = 50 μm. **c** Metacestode sections were stained with PAS. PAS stain presented a strongly PAS-positive basophilic laminated layer. Scale bar = 50 μm. **d** Different concentrations of thiacloprid or PZQ against *E. multilocularis* metacestodes. The thiacloprid or PZQ were tested by PGI assay on *E. multilocularis* metacestodes in a concentration series ranging from 0.6 to 160 μM in triplicates. Treatment with 0.1% Triton X-100 served as a positive control and was set as 100%. The data are presented as the mean ± SD. **e** Metacestode vesicle morphology after thiacloprid or PZQ treatment. Metacestode vesicles collapsed after 5 μM thiacloprid or PZQ treatment. **f** SEM of *E. multilocularis* metacestodes incubated in vitro with thiacloprid or PZQ. GL: germinal layer

We successfully cultured *E. multilocularis* metacestode vesicles in vitro (Additional file 4: Figure S4). In the RT-PCR analysis, there was no amplification of mouse GAPDH in metacestode vesicles and germinal cells, thus confirming the absence of host cells, while the *E. multilocularis* GAPDH specific gene was amplified from metacestode esicles and germinal cells (Fig. 1a). Metacestode vesicles showed obvious germinal layer by HE staining (Fig. 1b). PAS staining showed positive laminated layer (Fig. 1c).

Seven neonicotinoids were initially screened in vitro on *E. multilocularis* metacestode vesicles at 20 μM. Only thiacloprid showed activity at 5 days and 12 days after PGI assay screening (Additional file 2: Figure S2). Subsequently, the metacestode vesicles were then treated with thiacloprid or PZQ at a series of concentrations (Fig. 1d). The EC_50_ of PZQ against *E. multilocularis* metacestode was 7.41 ± 2.09 μM by the PGI assay. Thiacloprid showed significant anti-metacestode activity. The EC_50_ value was determined to be 4.54 ± 1.10 μM by the PGI assay. Morphological alterations of metacestode vesicles resulted from thiacloprid were observed under a light microscope (Fig. 1e). After 5 days of treatment with 5 μM thiacloprid, the vesicles exhibited contraction and collapse. The in vitro effect of thiacloprid was further confirmed by SEM (Fig. 1f). The *E. multilocularis* metacestode vesicles in the untreated group showed a typical intact structure: tegument was tightly attached to the germinal layer. Metacestode incubated with 5 μM thiacloprid or PZQ for 5 days showed noticeable damage: only residual cellular material was observed in many parts of germinal layer tissue.

### Cytotoxicity measurements of thiacloprid on HFF and RH cells

**Fig. 2:**
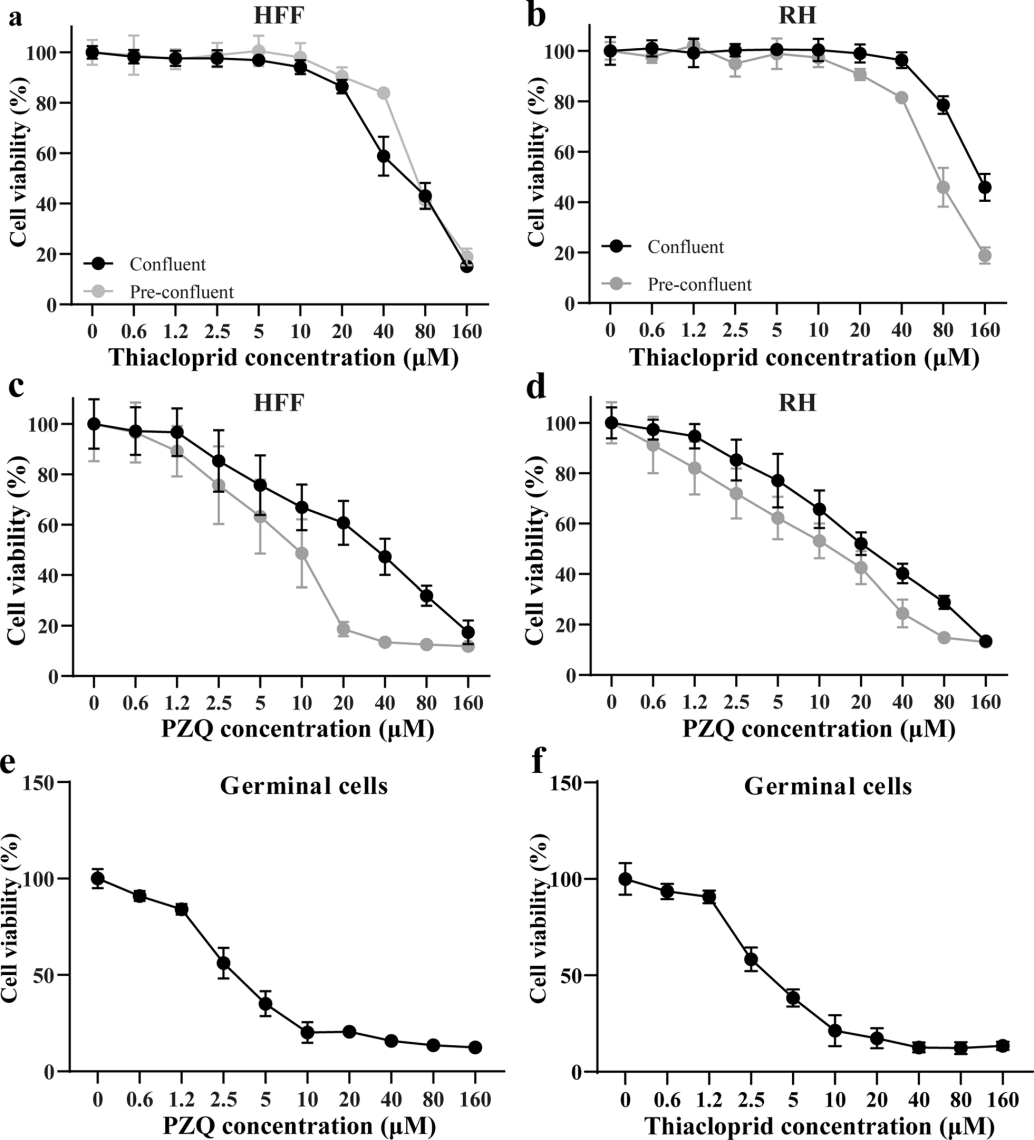
Cytotoxicity of thiacloprid or PZQ on mammalian cells. Viability measurements on HFF and RH cells employed alamarBlue assay, while for *E. multilocularis* germinal cells viability was assessed by CellTiter-Glo assay. The data are presented as mean ± SD obtained from three independent experiments. **a**, **b** In vitro activity of thiacloprid against HFF and RH cells. **c**, **d** In vitro activity of PZQ against HFF and RH cells. **e** Cytotoxicity of thiacloprid on *E. multilocularis* germinal cells. **f** Cytotoxicity of PZQ on *E. multilocularis* germinal cells

Cytotoxicity was assessed by Alamar Blue assay for thiacloprid or PZQ using confluent and pre-confluent cultures of HFF and RH cells (Fig. 2a, b). The IC_50_ values of thiacloprid on confluent and pre-confluent HFF were 68.73 ± 9.71 μM and 64.84 ± 5.79 μM, respectively, while the IC_50_ values for confluent and pre-confluent RH cells were 91.36 ± 1.33 μM and 74.34 ± 10.85 μM, respectively. The IC_50_ values of PZQ on confluent and pre-confluent HFF were 53.21 ± 11.05 μM and 6.71 ± 1.88 μM, respectively (Fig. 2c). The IC_50_ values of PZQ on confluent and pre-confluent RH cells were 25.29 ± 6.32 μM and 11.63 ± 3.52 μM, respectively (Fig. 2d). Thiacloprid or PZQ was less toxic to HFF and RH cells than to *E. multilocularis* metacestodes.

### Effect of thiacloprid on the viability of *E. multilocularis* germinal cells

Germinal cells were isolated by trypsin digestion from in vitro-cultured metacestode vesicles (Additional file 6: Figure S4). The CellTiter-Glo assay was used to determine the effect of thiacloprid or PZQ on the germinal cells. The IC_50_ of PZQ against germinal layer cells was 2.59 ± 0.33 μM (Fig. 2e). Thiacloprid significantly inhibited germinal cell viability (Fig. 2f). More than 10 μM of thiacloprid resulted in a significant reduction of viable cells with an inhibition rate of more than 80%. At 2.5 and 5 μM, the cell viability rates of thiacloprid on germinal cells were 58.29 ± 6.09% and 38.27 ± 4.47%, respectively. The IC_50_ of thiacloprid against germinal cells was 2.89 ± 0.34 μM.

### Transmission electron microscopy visualization of the effects of thiacloprid treatment in *E. multilocularis* metacestodes

**Fig. 3:**
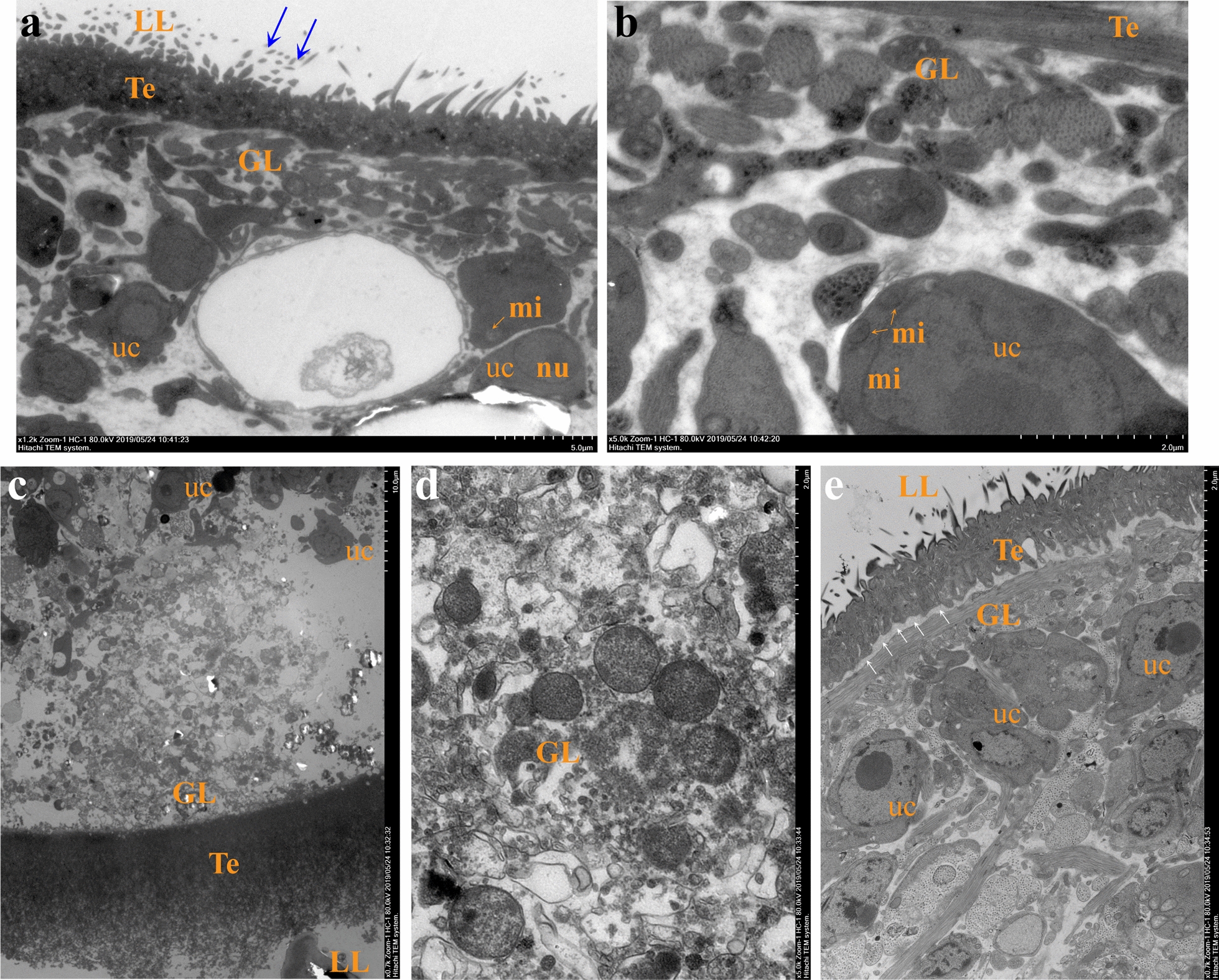
Transmission electron microscopy of *E. multilocularis* metacestodes exposed to 5 μM thiacloprid for 5 days. **a**, **b** The normal metacestode structure. LL: laminated layer; Te: tegument; GL: germinal layer; uc: stem cell with large nucleus and nucleolus; mi: mitochondria. The blue arrows show microtriches. Scale bars in (**a**) and (**b**) are 5 μm and 2 μm, respectively. **c**, **d** The germinal layer structural damage. Scale bars in (**c**) and (**d**) are 10 μm and 2 μm, respectively. **e** The germinal layer was separated from the tegument (white arrow). Scale bar = 2 μm

Based on the determination of the anti-metacestode effect of thiacloprid, the ultrastructural effect of thiacloprid against *E. multilocularis* metacestode was further confirmed by TEM. Untreated *E. multilocularis* metacestode vesicles have typical morphological characteristics (Fig. 3a, b): the outer of the parasite tissue is composed of laminated layers, which are rich in carbohydrates and separate the parasitic tissue from the surrounding host tissue; the inner surface of the laminated layer is the tegument with numerous microtriches protruding into the laminated layer. The germinal layer, which adheres to the capsule, contains a variety of cell types, including muscle cells, subtegumentary cytons, connective tissue, and undifferentiated stem cells. After 5 μM thiacloprid treatment, obvious ultrastructural damage was observed. The microtriches fell off obviously and the tegument became loose (Fig. 3c). The germinal layer structure was obviously destroyed and the cell structure was disordered (Fig. 3d). Part of the metacestode vesicle has observed germinal layer and tegument separation (Fig. 3e).

### In vitro activity of thiacloprid against *E. multilocularis* protoscoleces

**Fig. 4:**
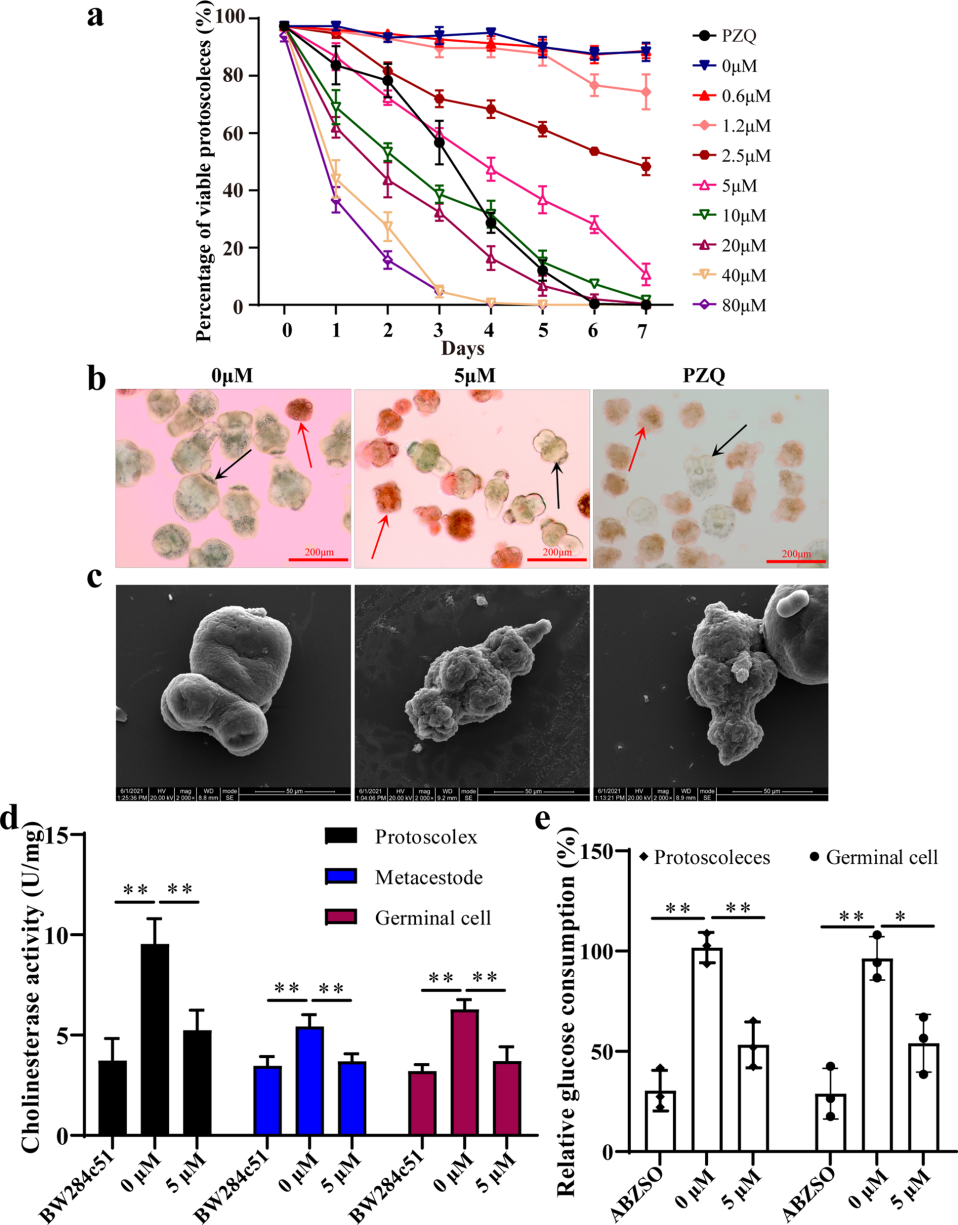
Thiacloprid against *E. multilocularis* protoscoleces and inhibited acetylcholinesterase activity. **a** Survival of protoscoleces after treatment with thiacloprid. The protoscoleces were exposed to thiacloprid for 7 days. To evaluate the survival of protoscoleces, a 0.1% eosin staining exclusion method was used daily. The positive control was 5 μM PZQ. The data are presented as mean ± SD obtained from three independent experiments. **b** Morphologies of protoscoleces after treatment with thiacloprid. After treatment with 5 μM thiacloprid for 4 days, eosin staining was used to observe the protoscoleces. The red arrow shows the dead protoscoleces and the black arrow shows the live protoscoleces. Scale bar = 200 μm. **c** SEM of *E. multilocularis* protoscoleces incubated in vitro with 5 μM thiacloprid for 4 days. **d** Thiacloprid inhibited acetylcholinesterase activity. BW284c51 was used as the positive control. The data are presented as mean ± SD obtained from five independent experiments. **e** Thiacloprid inhibits glucose consumption. The protoscoleces or germinal cells were treated with 5 μM thiacloprid, and relative glucose utilization was determined. The data are presented as mean ± SD obtained from three independent experiments. *P < 0.05 and **P < 0.01

After thiacloprid treatment at 5 μM for 4 days, 47.33 ± 4.04% of protoscoleces were dead, while there was no significant effect on their viability after 1.2 μM thiacloprid treatment (Fig. 4a). The dead protoscoleces were distinguished from the viable protoscoleces by eosin exclusion and observed under a light microscope (Fig. 4b). Furthermore, ultrastructural damage was revealed by SEM in the protoscoleces treated with thiacloprid at 5 μM for 4 days compared with the protoscoleces without treatment (Fig. 4c). There was no significant change in the untreated protoscoleces. In contrast, after thiacloprid treatment, the surface of the protoscoleces was significantly contracted, and no obvious microvilli were observed. The positive control PZQ showed the same performance as thiacloprid treatment (Fig. 4c).

### Thiacloprid inhibits acetylcholinesterase activity and glucose absorption

Previous studies have shown that the inhibition of parasite acetylcholinesterase activity can suppress the absorption of host-derived glucose [36]. Our study confirmed the presence of acetylcholinesterase in the *E. multilocularis* protoscoleces, metacestodes and germinal cells (Additional file 7: Table S3). After thiacloprid treatment, acetylcholinesterase activity in protoscoleces, metacestode vesicles and germinal cells was suppressed (Fig. 4d). Positive control BW284c51 also inhibited acetylcholinesterase activity. Glucose consumption in *E. multilocularis* protoscoleces and germinal cells was also measured. The viability of protoscoleces and germinal cells was not affected by thiacloprid treatment for 1 h (Additional file 8: Figure S5). Both the protoscoleces and germinal cells exposed to thiacloprid showed reduced uptake of 2-DG. These results indicate that thiacloprid has the effect of inhibiting glucose uptake in protoscoleces and germinal cells (Fig. 4e).

### Toxicity evaluation of thiacloprid in vivo

**Fig. 5:**
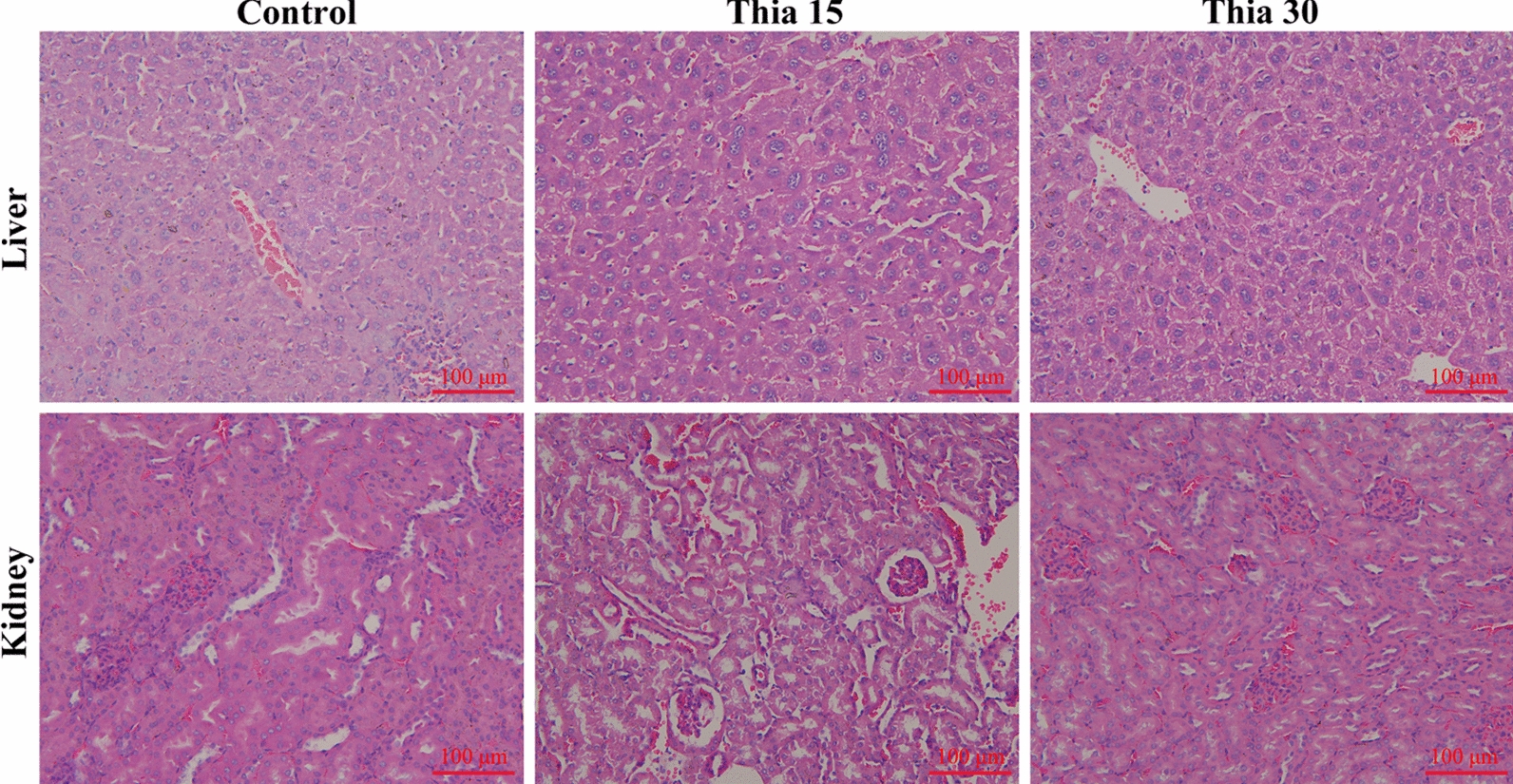
Histopathological examination of liver and kidneys in thiacloprid-treated mice. Control tissues and thiacloprid-treated tissues were observed microscopically. Scale bar = 100 μm

Adverse reactions of a drug can greatly limit its clinical application. Here, we further analyzed the in vivo toxicity of thiacloprid. For this, BALB/c mice were treated with thiacloprid for 6 weeks. The levels of ALT, AST, TBIL, DBIL, IBIL, TP, ALB, ALP, CREA and BUN in serum and the levels of WBC, Hb and PLT in whole blood are shown in Table 1. There was no significant difference in the above biochemical and hematological parameters between the thiacloprid treated group and the control group. These tests showed that thiacloprid had no significant effect on liver and kidney function in mice. Furthermore, the liver and kidneys of mice were subjected to histopathological examination after HE staining (Fig. 5). The livers and kidneys of mice treated with thiacloprid did not show obvious histopathological changes or injury.

**Table 1:** Serum biochemical findings and blood cell analysis in BALB/c mice treated with thiacloprid for 6 weeks (*n* = 6)

Balb/c mice	Control	Thia 15	Thia 30
Alanine aminotransferase (U/L)	65.67 ± 6.53	66.00 ± 5.33	67.50 ± 5.01
Aspartate aminotransferase (U/L)	198.20 ± 28.15	164.70 ± 36.71	180.50 ± 45.71
Alkaline phosphatase (U/L)	84.33 ± 4.46	57.67 ± 5.43^*^	58.50 ± 7.82*
Total protein (g/L)	63.30 ± 2.41	65.90 ± 4.76	65.82 ± 4.39
Globulin (g/L)	34.80 ± 2.83	36.67 ± 4.71	37.77 ± 4.81
Albumin (g/L)	28.50 ± 1.57	28.95 ± 2.35	29.72 ± 1.49
Total bilirubin (μM/L)	0.66 ± 0.22	0.60 ± 0.10	0.59 ± 0.16
Direct bilirubin (μM/L)	1.10 ± 0.05	0.13 ± 0.05	0.19 ± 0.16
Indirect bilirubin (μM/L)	0.51 ± 0.25	0.44 ± 0.17	0.42 ± 0.19
Blood urea nitrogen (mmol/L)	9.31 ± 1.02	7.23 ± 1.87^*^	7.15 ± 0.47^*^
Creatinine (μM/L)	16.07 ± 3.43	16.08 ± 1.87	15.18 ± 2.44
RBC (10^12^/L)	10.97 ± 1.26	11.39 ± 1.48	9.58 ± 0.82
Hemoglobin (g/L)	214.30 ± 11.78	203.00 ± 11.15	206.0 ± 9.93
WBC (10^9^/L)	12.92 ± 0.18	11.69 ± 1.30	12.72 ± 1.75
PLT (10^9^/L)	673.00 ± 120.80	613.70 ± 145.00	670.80 ± 150.20

*P < 0.05 vs. control

### Effect of thiacloprid against *E. multilocularis* metacestodes in vivo

**Fig. 6:**
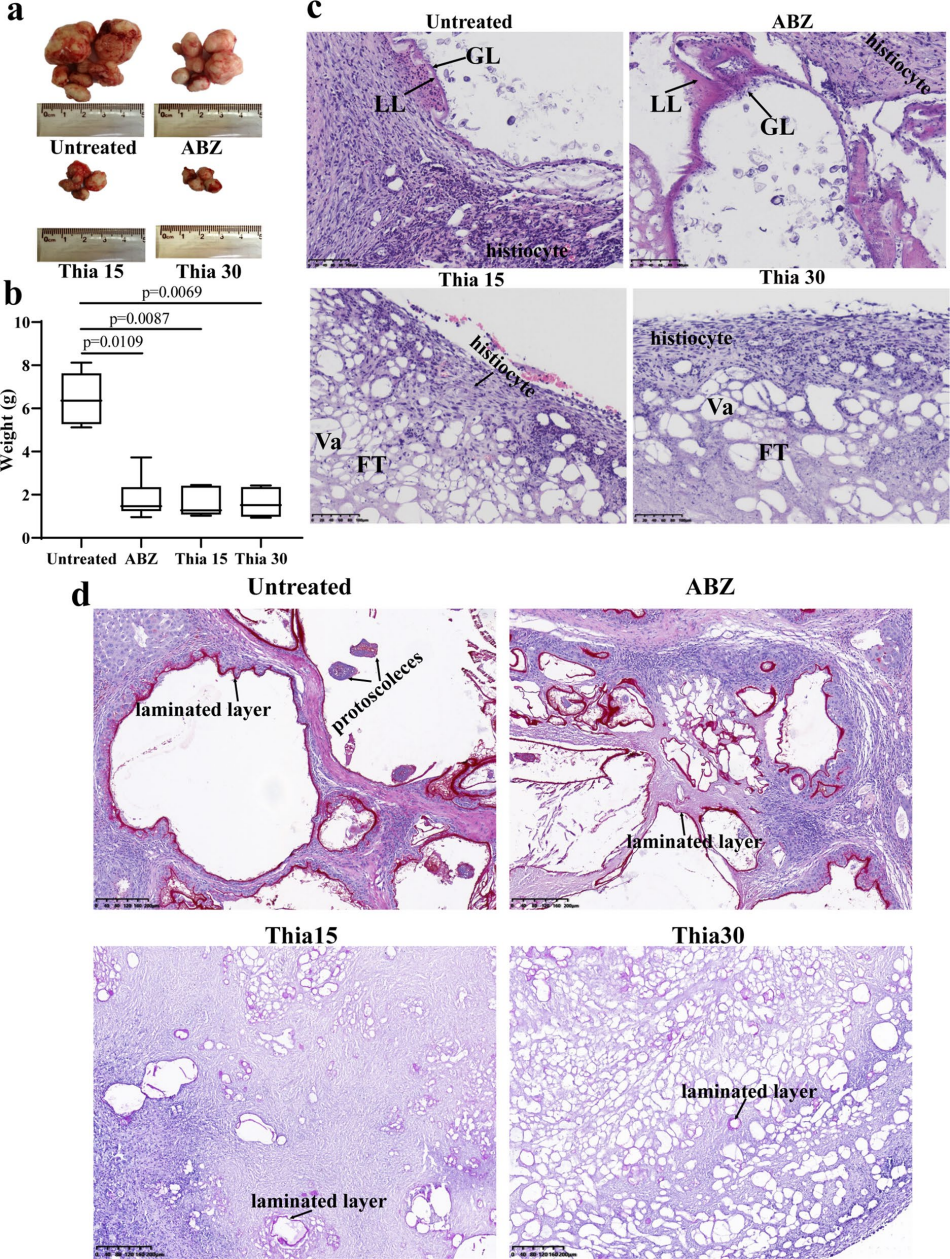
In vivo activity of thiacloprid against *E. multilocularis* metacestodes. Two weeks after infection of BALB/c mice with metacestode tissues, the mice received the intragastric administration of thiacloprid for 6 weeks. The positive control was 100 mg/kg ABZ. **a** The images of metacestodes resected from different treatment groups. **b** Parasite weight in different treatment groups. The data are presented as mean ± SD from seven independent experiments. **c** HE staining of *E. multilocularis* metacestodes from different treatment groups. Scale bar = 100 μm. LL: laminated layer; FT: fibrous tissue; Va: vacuole. **d** Laminated layer PAS staining. The section shows PAS-positive laminated layer of *E. multilocularis* metacestodes. Scale bar = 200 μm

An in vivo experiment was performed to investigate the therapeutic effect of thiacloprid. After 6 weeks of treatment, each metacestode in mice presented with polycystic growth, and representative metacestodes are presented in Additional file 9: Figure S6 and Fig. 6a. The metacestode weight data was not normally distributed according to Shapiro–Wilk test (*W* = 0.787; *P* = 0.000). Kruskal–Wallis analysis indicated a significant reduction of the wet weights of the metacestodes in the Thia15 group (1.63 ± 0.62 g), Thia30 group (1.63 ± 0.61 g) and ABZ group (1.85 ± 0.94 g) compared with the control group (6.49 ± 1.18 g) (Fig. 6b). Thiacloprid treatment seemed to be more effective than ABZ, as there was no significant difference in the weight of their metacestodes. Thiacloprid also did not show dose-dependent tolerance. For pathological observation, the center of the lesions in the respective group presented caseous necrosis (Additional file 10: Figure S7). Considerable vacuoles could be observed in the metacestode after treatment with thiacloprid (Fig. 6c). However, germinal cells could still be observed in the ABZ group and untreated group (Fig. 6c). In addition, in the thiacloprid-treated group, only a few laminated layers were observed by PAS staining (Fig. 6d). Since spleen enlargement was observed after thiacloprid treatment, changes in T lymphocyte subsets and cytokines were further investigated.

### Thiacloprid regulates lymphocyte subset

**Fig. 7:**
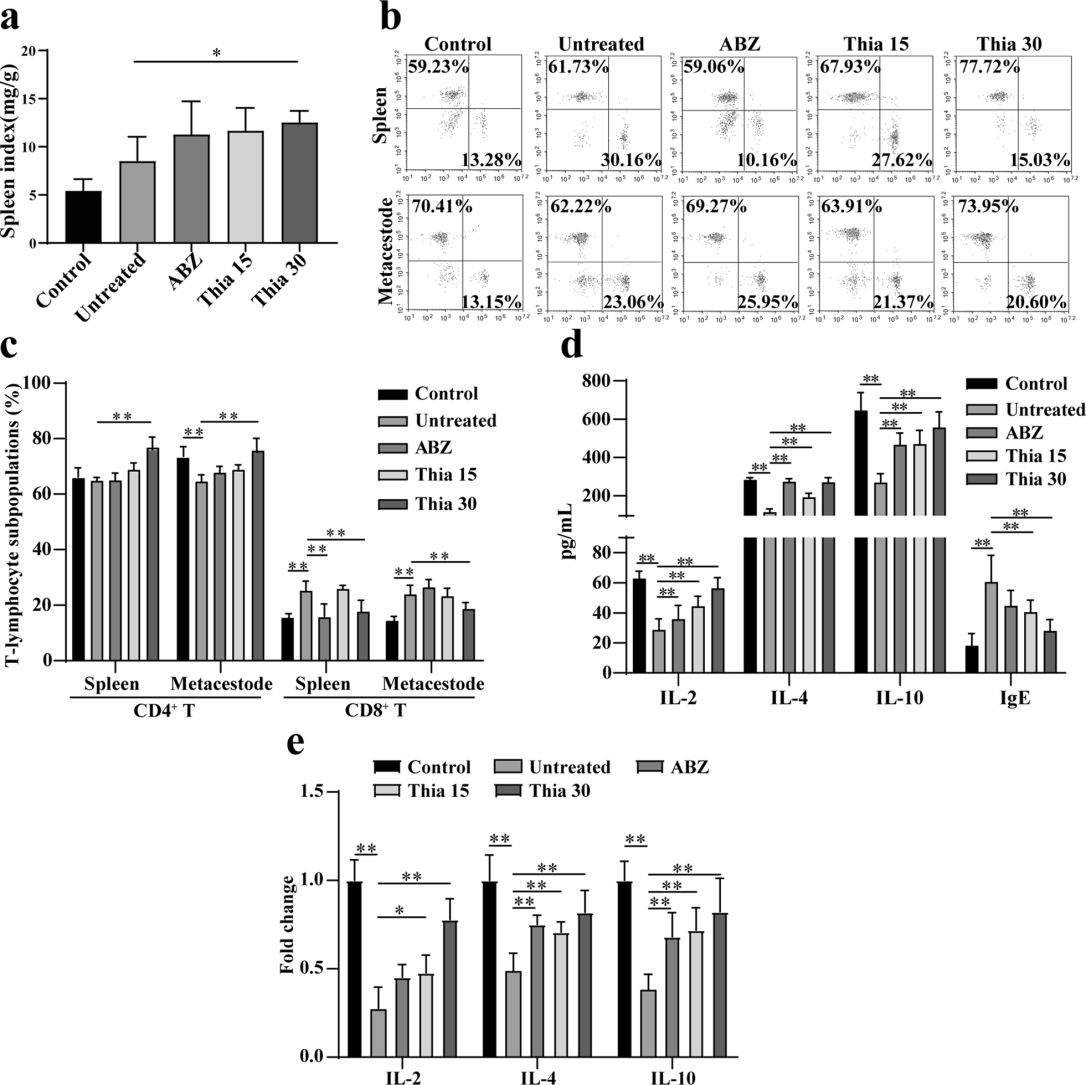
T lymphocyte subsets and cytokine analysis. **a** Spleen index of mice treated with thiacloprid. Data are presented as mean ± SD obtained from seven independent experiments. **b**, **c** Variations in lymphocyte subsets in metacestodes and spleen. Metacestodes and spleen lymphocytes stained with FITC-CD3, PE-CD4 and APC-CD8. CD3^+^ CD4^+^ and CD3^+^ CD8^+^ T cells were detected via flow cytometry. Data are presented as mean ± SD obtained from six independent experiments. **d** The levels of IL-2, IL-4, IL-10 and IgE cytokines after the thiacloprid treatment. The data are presented as mean ± SD obtained from seven independent experiments. **e** Cytokine levels in metacestode microcyst fluid. The data are presented as mean ± SD obtained from six independent experiments

In the isolation of metacestodes in mice, we observed enlarged spleens, and the spleen index of mice increased after treatment with thiacloprid (Fig. 7a). Since cellular immunity is critical to AE, we further analyzed the effect of thiacloprid on T lymphocyte subsets in the metacestodes and spleen. According to the analysis of blood cells, the percentage of lymphocytes increased, and the percentage of eosinophils and neutrophils was downregulated after treatment with thiacloprid (Additional file 11: Table S4). Furthermore, after treatment with thiacloprid, CD4^+^ T lymphocytes increased and CD8^+^ T lymphocytes decreased as compared with the untreated group in the metacestodes and spleen (Fig. 7b, c). Moreover, the infiltration of neutrophils in the metacestodes was reduced, while there were more infiltration of plasma cells, lymphocytes and macrophages (Additional file 12: Figure S8).

### Thiacloprid enhances the level of anti-AE cytokines in serum

To further assess the immune effects induced by thiacloprid, cytokine levels were detected by ELISA. *Echinococcus multilocularis* metacestode infection caused the expression of IL-2, IL-4 and IL-10 to be downregulated, and the expression of IgE to be upregulated. Thiacloprid treatment upregulated the expression of IL-2, IL-4 and IL-10 and downregulated the expression of IgE compared with the untreated group (Fig. 7d). ABZ led to consistent variations. Moreover, the expression of IL-2, IL-4 and IL-10 in microcyst fluid was upregulated after thiacloprid treatment (Fig. 7e).

### Collagen deposition in the host–lesion microenvironment

**Fig. 8:**
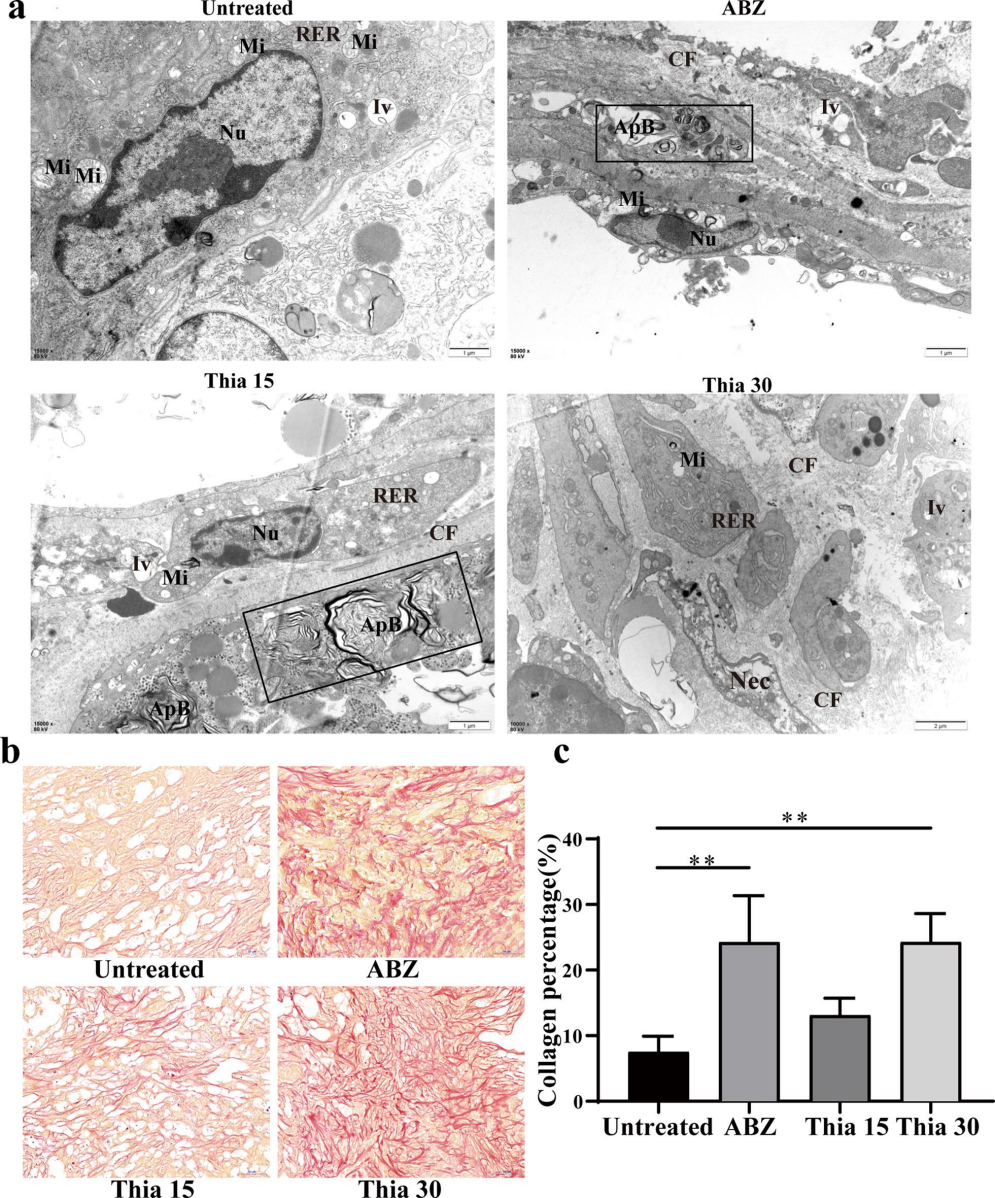
The host–lesion microenvironment structure was observed after in vivo thiacloprid treatment. **a** TEM observation of the host–lesion microenvironment. Nu: nucleus; Nec: necrosis; Iv: intracytoplasmic vacuoles; RER: rough endoplasmic reticulum; ApB: Autophagy body; CF: collagenous fibre. **b** Picrosirius red staining. Collagen fibres appear red after staining. **c** Quantitative analysis of collagen fiber content. The data are presented as the mean ± SD of six experiments. **P < 0.01

TEM was used to observe the ultrastructural changes of the host–lesion microenvironment (Fig. 8a). Abundant mitochondria, endoplasmic reticulum and ribosomes could be seen in the untreated group, and rough endoplasmic reticulum expanded obviously. In the ABZ group, autophagosomes appeared; there were many vacuoles in the cytoplasm; collagen fibers were deposited in the extracellular matrix. In the microenvironment of the Thia15 group, mitochondrial cristae ruptured or disappeared and autophagosomes appeared, and a large number of collagen fibers were deposited in the intercellular substance. In the microenvironment of the Thia30 group, cell necrosis was observed. Mitochondrial cristae disappeared; many vacuoles were found in the cytoplasm, and abundant collagen fibres were deposited in the intercellular substance. Sirius red staining further confirmed collagen fiber deposition in the host–lesion microenvironment after thiacloprid or PZQ treatment (Fig. 8b, c). The results show that thiacloprid or ABZ can promote autophagy in the host–host microenvironment, especially collagen fiber deposition.

### Thiacloprid inhibits the activity of matrix metalloproteinases (MMPs)

**Fig. 9:**
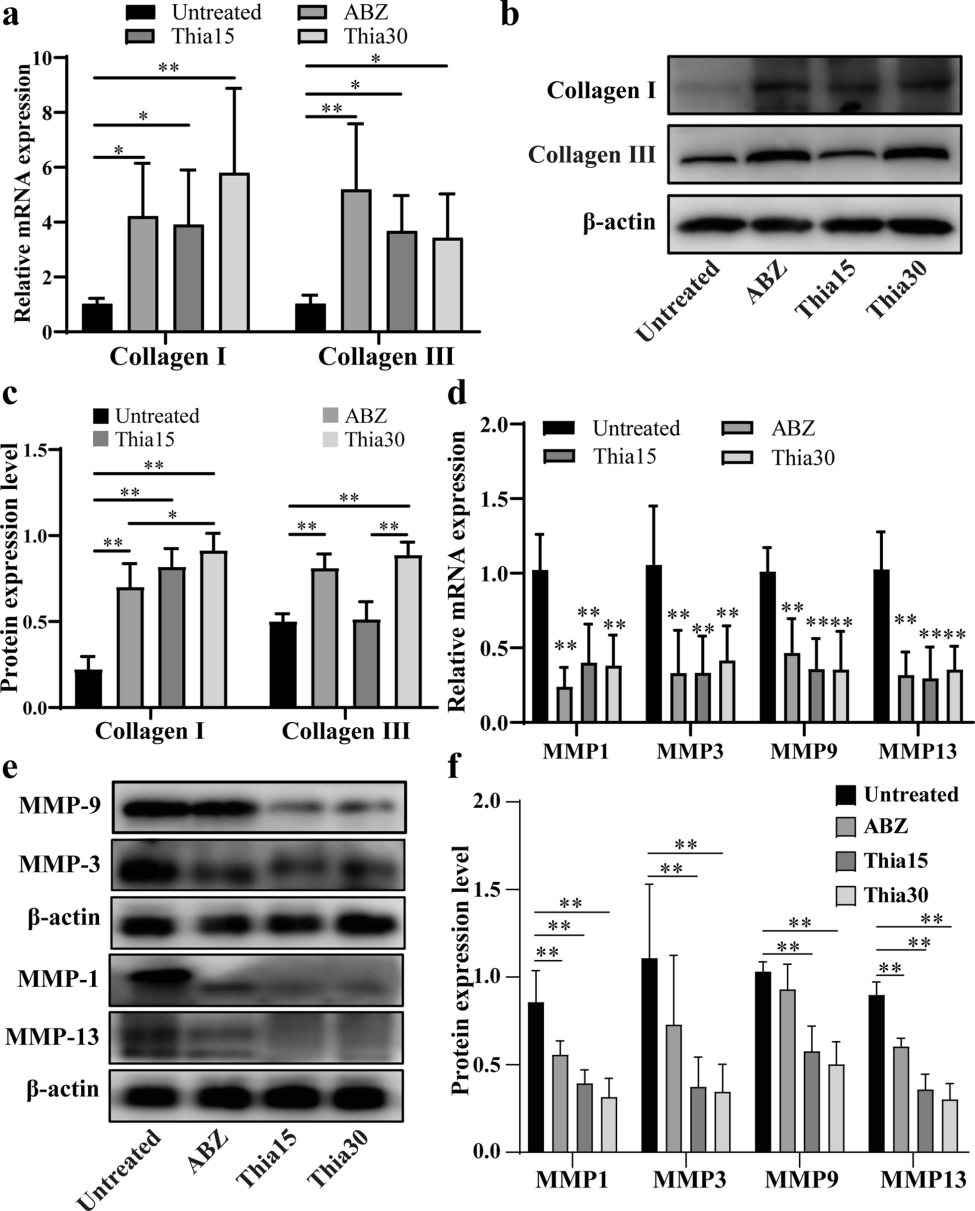
Thiacloprid inhibits MMP activity in the host–lesion microenvironment. **a** Quantitative PCR analysis of collagen I and III expression in the host–lesion microenvironment. The data are presented as the mean ± SD of six experiments. **b** Western blotting was used to detect the protein expression of collagen I and III. **c** Quantitative analysis of collagen I and III protein levels. The data are presented as the mean ± SD of five experiments. **d** Quantitative PCR analysis of MMP expression in the host–lesion microenvironment. The data are presented as the mean ± SD of six experiments. **e** Western blotting was used to detect the protein expression of MMPs. **f** Quantitative analysis of MMP protein levels. The data are presented as the mean ± SD of five experiments. *P < 0.05 and **P < 0.01

MMPs are the main factors leading to collagen degradation; thus, we further studied the effect of thiacloprid on MMP production. After thialoprid or ABZ treatment, mRNA and protein expression levels of collagen I and III in the host–lesion microenvironment were increased (Fig. 9a–c). We then evaluated the mRNA expression levels of related MMPs in the microenvironment (Fig. 9d). After thiacloprid or ABZ treatment, the mRNA expression of MMP1, MMP3, MMP9 and MMP13 in the microenvironment was significantly inhibited. In addition, the protein expression levels of MMP1, MMP3, MMP9 and MMP13 were decreased after thiacloprid or ABZ treatment (Fig. 9e, f). The results demonstrate that thiacloprid can reduce the breakdown of extracellular collagen by inhibiting the expression of MMPs.

## Discussion

Echinococcosis remains a major public health problem worldwide, seriously affecting the health of people in pastoral areas and causing substantial social problems and economic losses to animal husbandry [37]. Current chemotherapy regimens rely on benzimidazole treatment. The role of drug therapy pre- and post-AE surgery is irreplaceable. However, it can only inhibit the growth and dispersion of metacestodes, but cannot kill the parasite [38]. Therefore, new drugs or lead compounds are needed.

Since the initial identification of members of the *E. multilocularis* nAchR family, few studies have explored the effects of echinococcus nAchR-targeting drugs on parasite growth and development [39]. In the present study, we screened the neonicotinoids in vitro for active compounds against *E. multilocularis* metacestodes using PGI assays. The glycolytic enzyme PGI is a moonlighting protein that has been shown to stimulate the proliferation of both parasite germinal cells and mammalian endothelial cells [40]. In recent years, in vitro models of *E. multilocularis* have been able to produce large-scale metacestode vesicles in favor of drug screening. Meanwhile, the fact that PGI is a prominent component of the vesicle fluid has been exploited for the development of the PGI screening assay, which enables quantitative screening for drugs that impair the structural integrity of metacestodes in such a way that vesicle fluid is released into the medium supernatant [29]. All the neonicotinoids acted on metacestode vesicles at a dose of 20 μM, but only thiacloprid showed activity. The EC_50_/IC_50_ value of thiacloprid anti-metacestode vesicle and germinal cell activity was 4.54 ± 1.10 μM and 2.89 ± 0.34 μM, which was significantly lower than the toxicity of thiacloprid to mammalian cells. This suggests that thiacloprid has potential therapeutic effects for further analysis. We observed by TEM that thiacloprid caused the germinal layer structure to be destroyed or the germinal layer separated from tegument. Although treatment with 5 μM thiacloprid for 4 days only resulted in the death of 47.33 ± 4.04% protoscoleces, the survival of germinal cells was found to be less than 40% at 5 μM. These results indicated that germinal cells were more sensitive to thiacloprid than protoscoleces. We also demonstrated that thiacloprid can affect protoscolex acetylcholinesterase activity. It has been reported that neonicotinoids inhibit the activity of acetylcholinesterase in worm [41]. With parasite acetylcholinesterase activity inhibited, the breakdown of acetylcholine will reduce the absorption of host-derived glucose [36]. Our results demonstrated that thiacloprid treatment reduced glucose absorption. Therefore, thiacloprid may play a parasitic role by inhibiting the glucose absorption of the protoscoleces.

The wet weight of metacestodes decreased significantly after thiacloprid treatment in mice, demonstrating an identical effect to albendazole [42]. The germinal layer was obviously destroyed after thiacloprid treatment. Thiacloprid caused a decrease in the number of eosinophils and neutrophils in the blood. Eosinophils are recognized as end-stage cells involved in protecting the host against parasitic infections [43]. Once the parasite dies or escapes host immunity, inflammation is mitigated [44]. No obvious eosinophil deposition was identified in the metacestode, whereas there was considerable lymphocyte, plasma cell and macrophage aggregation. The effect of albendazole on eosinophils in parasite infection remains controversial [42, 45]. Plasma cells are capable of secreting immunoglobulin and penetrating the metacestode wall to exert an anti-echinococcosis effect. White blood cells are involved in cell differentiation, inflammatory response expansion, and even innate and adaptive immune regulation [46].

Anti-echinococcosis immunity is dominated by T cell-mediated cellular immune responses [47]. In the state of chronic infection, there are numerous activated CD8^+^ T lymphocytes in the peripheral blood [48], and CD8^+^ T lymphocytes cause immunosuppression in AE [49]. CD4^+^ T lymphocyte-mediated cellular immunity is required to resist *E. multilocularis* infection [50]. The use of CD4-deficient and CD8-deficient mice proves that CD4^+^ T lymphocyte-mediated cellular immunity is necessary to resist *E. multilocularis* infection [50]. Thiacloprid increased the ratio of CD4^+^ T cells in metacestodes and spleen, while downregulating the ratio of CD8^+^ T cells, which resulted in an increase in the ratio of CD4^+^/CD8^+^ T lymphocytes. This demonstrated that the anti-echinococcosis effect of thiacloprid in vivo may also enhance the cellular immune response. Thiacloprid reversed the immunosuppression after *E. multilocularis* metacestode infection. However, there is an imbalance between the activation of Th1 and Th2 cell immune response during the occurrence of echinococcosis, resulting in differences in cytokine secretion. In the host immune response to parasite infection, IL-4 is crucial to the protective immunity against extracellular parasite infection [51, 52]. IL-2 is recorded as a critical cytokine in positive immune regulation [53]. Thiacloprid led to elevated expression of IL-2, IL-4, IL-10 and downregulated the expression of IgE in mice with secondary infection of AE, and ABZ exerted a consistent effect. The application of thiacloprid is conducive to mitigating the immunosuppression of *E. multilocularis* metacestode infection. However, as indicated by excessive IL-10, the immune function of T-lymphocytes is in an inhibited state, which is conducive to the survival of parasites [54]. Thus it could be speculated that IL-10 may play an anti-echinococcosis role by downregulating VEGF expression and inhibiting angiogenesis in the metacestodes [55]. Although certain insights into immunization against echinococcosis have been gained, many immune mechanisms remain unclear, which should be researched in greater depth.

The most prominent feature of the host–lesion microenvironment is the effective accumulation of ECM, the main component of which is collagen [4, 6]. Our study confirmed that the deposition of collagen I and III in the host–lesion microenvironment increased after treatment with thiacloprid, which may be beneficial in promoting fibrosis and calcification of metacestodes. MMPs are the main enzymes for collagen degradation in ECM [56]. To evaluate whether the increase in collagen is accompanied by changes in MMPs, we detected MMPs related to the degradation of type I and III collagen and found that thiacloprid induced a decrease in MMP-1, MMP-3, MMP-9 and MMP-13 protein expression levels in the host–lesion microenvironment. MMPs are a group of endogenous proteases that rely on zinc and calcium to catalyze their activity. They are secreted in the extracellular space and degrade all molecular components of ECM [57]. Therefore, our results demonstrated that the promotion of collagen production by thiacloprid is probably related to the weakening of MMP activity.

The advantage of thiacloprid over nicotine lies in its lower toxicity. In fact, thiacloprid has been widely adopted to control surface parasites in cats and dogs [58]. Thiacloprid shows low toxicity to mammalian cells. Previous studies found that thiacloprid treatment with more than 108 mg/kg induced obvious hepatocellular hypertrophy and cytoplasm degeneration in mice [59]. Moreover, thiacloprid had no significant biochemical or hematological effects, and had no effect on liver or kidney morphology. This indicates that thiacloprid at concentrations below at least 30 mg/kg does not cause significant in vivo toxicity. Previous studies have shown that 22.5 mg/kg thiacloprid can cause hepatocellular necrosis and hydropic degeneration [35], and the reason for the inconsistent results may be related to the fact that the hypoxic environment in which we live reduces the absorption of drugs. Over the past few years, neonicotinoids have been found to be promising lead compounds for developing reversible cholinesterase inhibitors against Alzheimer’s disease [60]. However, although thiacloprid has low toxicity, high doses of thiacloprid still impact bone marrow erythrocytes and chromosomes to a certain extent [61]. In addition, neonicotinoids do not easily cross the blood–brain barrier, so thiacloprid in its current form is not suitable for the treatment of cerebral echinococcosis.

## Conclusions

Thiacloprid was identified from seven neonicotinoids with potent in vitro activity against *E. multilocularis* metacestodes. In addition, we demonstrated that thiacloprid inhibits metacestode and germinal cells acetylcholinesterase activity. Thiacloprid inhibited metacestode growth in vivo. Thiacloprid enhanced the activity of CD4^+^T lymphocytes, promoted the expression of anti-echinococcosis related cytokines in serum, and promoted host–lesion microenvironment collagen deposition. This study underlines the potential for the development of thiacloprid as an anti-*E. multilocularis* drug.

## Supplementary Information

**Additional file 1: Figure S1.** Chemical structure and functional groups of neonicotinoids. **a** Chemical structure of neonicotinoids. Chemical structure obtained from PubChem database. **b** Functional group information of neonicotinoids.

**Additional file 2: Figure S2.** Screening of the neonicotinoids on *E. multilocularis* metacestodes in vitro. Confirmation of active drugs by testing at 20 μM in triplicates. Relative PGI release as assessed by PGI-assay is shown. 100% PGI release was defined as the release upon treatment with the positive control 0.1% Triton-100. Drugs were considered as active if they exceeded 20% relative PGI release (dashed line).

**Additional file 3: Figure S3.** Isolation of metacestodes from Mongolian gerbil. The black arrows show the protoscoleces.

**Additional file 4: Table S1.** Specific primer sequences.

**Additional file 5: Table S2.** Antibody information.

**Additional file 6: Figure S4.** Metacestode vesicle (**a**) and germinal cells (**b**) cultured in vitro.

**Additional file 7: Table S3.***Echinococcus multilocularis* cholinesterase activity.

**Additional file 8: Figure S5.** The cell viability of protoscoleces and germinal cells treated with thiacloprid for 1 h. **a** Survival of protoscoleces after treatment with thiacloprid. To evaluate the survival of protoscoleces, 0.1% eosin staining exclusion method was used. **b** Mitochondrial probe labeling of living cells.

**Additional file 9: Figure S6.** Metacestode in the abdominal cavity of mice. The black arrows showed metacestodes.

**Additional file 10: Figure S7.** The center of the metacestodes showed caseous necrosis. HE staining showed caseous necrosis in the centre of the metacestode (scale bar = 400 μm). There is no structural granular and no residual shadow of the original tissue structure.

**Additional file 11: Table S4.** Blood cell analysis of mice infected with *E. multilocularis* treated with thiacloprid (*n* = 6).

**Additional file 12: Figure S8.** Immune cell infiltration in host tissue surrounding metacestode. After thiacloprid treatment, infiltration of neutrophils decreased (**a**) and infiltration of plasma cells, lymphocytes and macrophages increased in host tissue surrounding metacestode (**b**). The red arrow shows the neutrophils, the black arrow shows the lymphocytes, the green arrow shows the eosinophils, the blue arrow shows the plasma cells, and the yellow arrow shows the macrophages.

## Data Availability

Data supporting the conclusions of this article are included within the article and its additional files.

## Abbreviations

AE: *Alveolar echinococcosis*
*E. multilocularis*: *Echinococcus multilocularis*
PGI: Phosphoglucose isomerase
HFF: Human foreskin fibroblast
RH: Reuber rat hepatoma
2-DG: 2-Deoxyglucose
ELISA: Enzyme-linked immunosorbent assay
IL: Interleukin
MMP: Matrix metalloproteinase
ABZ: Albendazole
MBZ: Mebendazole
nAchRs: Nicotinic acetylcholine receptors
DMEM: Dulbecco’s modified Eagle medium
FBS: Fetal bovine serum
DMSO: Dimethyl sulfoxide
GAPDH: Glyceraldehyde-3-phosphate dehydrogenase
RT-PCR: Reverse transcription polymerase chain reaction
cDNA: Complementary DNA
PZQ: Praziquantel
EC_50_: Median effective concentration
IC_50_: Median inhibitory concentration
PBS: Phosphate-buffered saline
ABZSO: Albendazole sulfoxide
HE: Hematoxylin–eosin
PAS: Periodic acid-Schiff
OD: Optical density
IgE: Immunoglobulin E
SEM: Scanning electron microscopy
TEM: Transmission electron microscopy
RT-qPCR: Reverse transcription quantitative polymerase chain reaction
mRNA: Messenger RNA
SDS-PAGE: Sodium dodecyl sulfate polyacrylamide gel electrophoresis
TBST: Tris-buffered saline Tween 20
