# Supplementary material for: In vitro and in vivo efficacy of thiacloprid against Echinococcus multilocularis
Source: Parasit Vectors. 2021 Sep 6;14:450. doi: 10.1186/s13071-021-04952-7 (PMC8419995; doi:10.1186/s13071-021-04952-7)
Supplement: Supplementary file 1 — Additional file 1: Figure S1. Chemical structure and functional groups of neonicotinoids. a Chemical structure of neonicotinoids. Chemical structure obtained from PubChem database. b Functional group information of neonicotinoids. [file 13071_2021_4952_MOESM1_ESM.docx]

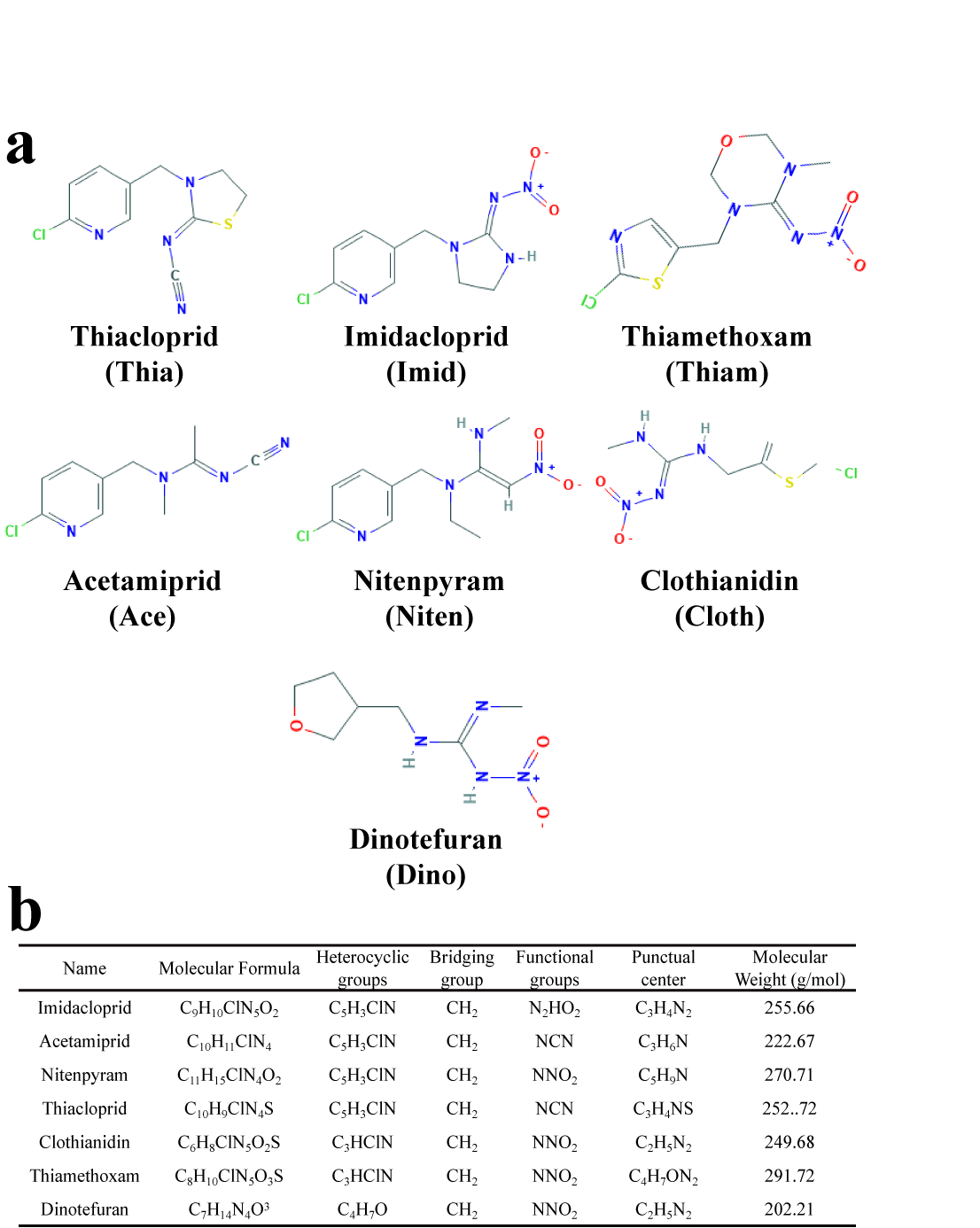


**Additional file 1: Figure S1. Chemical structure and functional groups of neonicotinoids. a** Chemical structure of neonicotinoids. Chemical structure obtained from Pubchem database. **b** Functional group information of neonicotinoids.
