# Supplementary material for: In vitro and in vivo efficacy of thiacloprid against Echinococcus multilocularis
Source: Parasit Vectors. 2021 Sep 6;14:450. doi: 10.1186/s13071-021-04952-7 (PMC8419995; doi:10.1186/s13071-021-04952-7)
Supplement: Supplementary file 2 — Additional file 2: Figure S2. Screening of the neonicotinoids on E. multilocularis metacestodes in vitro. Confirmation of active drugs by testing at 20 μM in triplicates. Relative PGI release as assessed by PGI-assay is shown. 100% PGI release was defined as the release upon treatment with the positive control 0.1% Triton-100. Drugs were considered as active if they exceeded 20% relative PGI release (dashed line). [file 13071_2021_4952_MOESM2_ESM.docx]

**
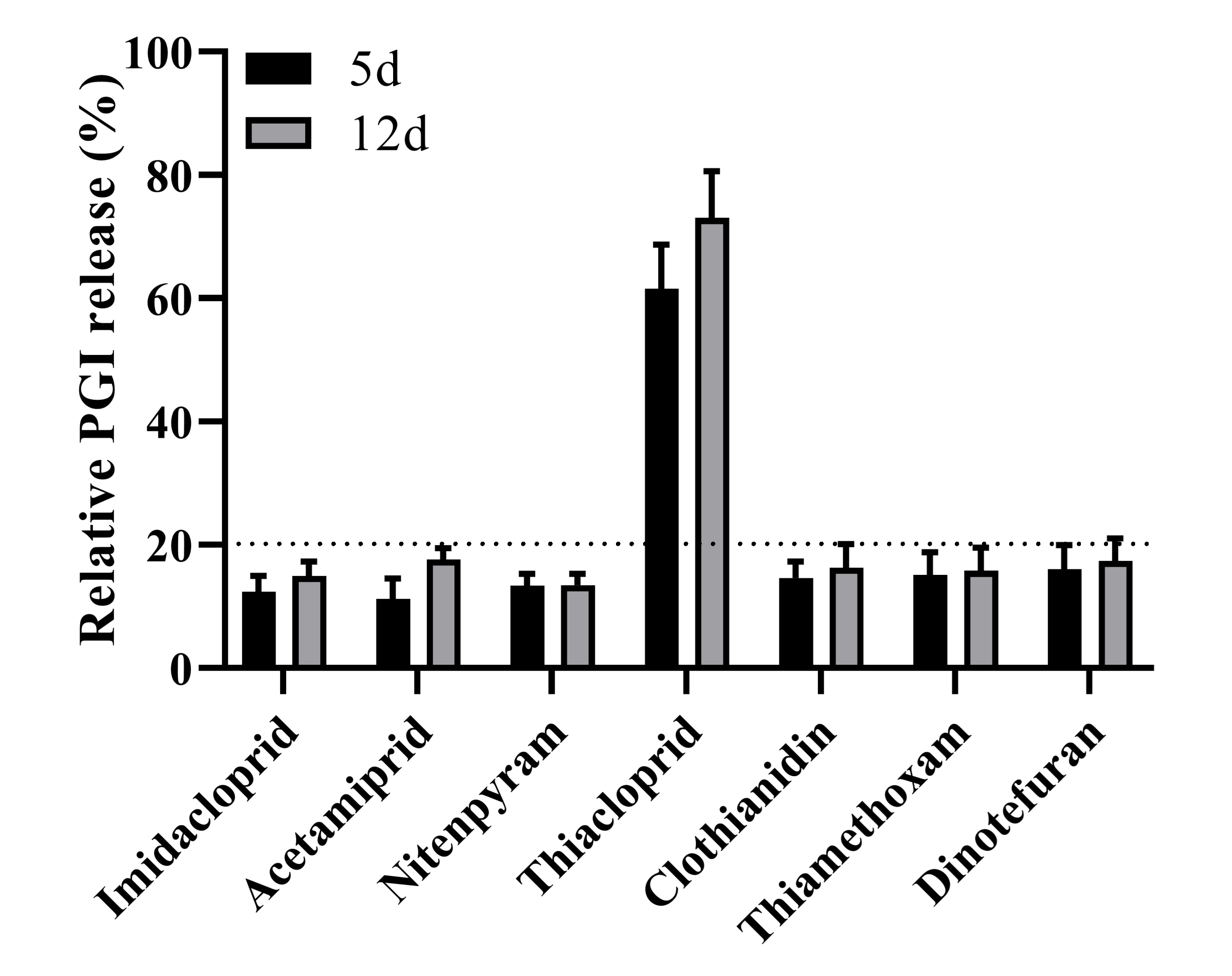
**

**Additional file 2: Figure S2. Screening of the neonicotinoids on *E. multilocularis* metacestodes *in vitro***. Confirmation of active drugs by testing at 20 μM in triplicates. Relative PGI release as assessed by PGI-assay is shown. 100% PGI release was defined as the release upon treatment with the positive control 0.1% Triton-100. Drugs were considered as active if they exceeded 20% relative PGI release (dashed line).
