# Supplementary material for: In vitro and in vivo efficacy of thiacloprid against Echinococcus multilocularis
Source: Parasit Vectors. 2021 Sep 6;14:450. doi: 10.1186/s13071-021-04952-7 (PMC8419995; doi:10.1186/s13071-021-04952-7)
Supplement: Supplementary file 3 — Additional file 3: Figure S3. Isolation of metacestodes from Mongolian gerbil. The black arrows show the protoscoleces. [file 13071_2021_4952_MOESM3_ESM.docx]

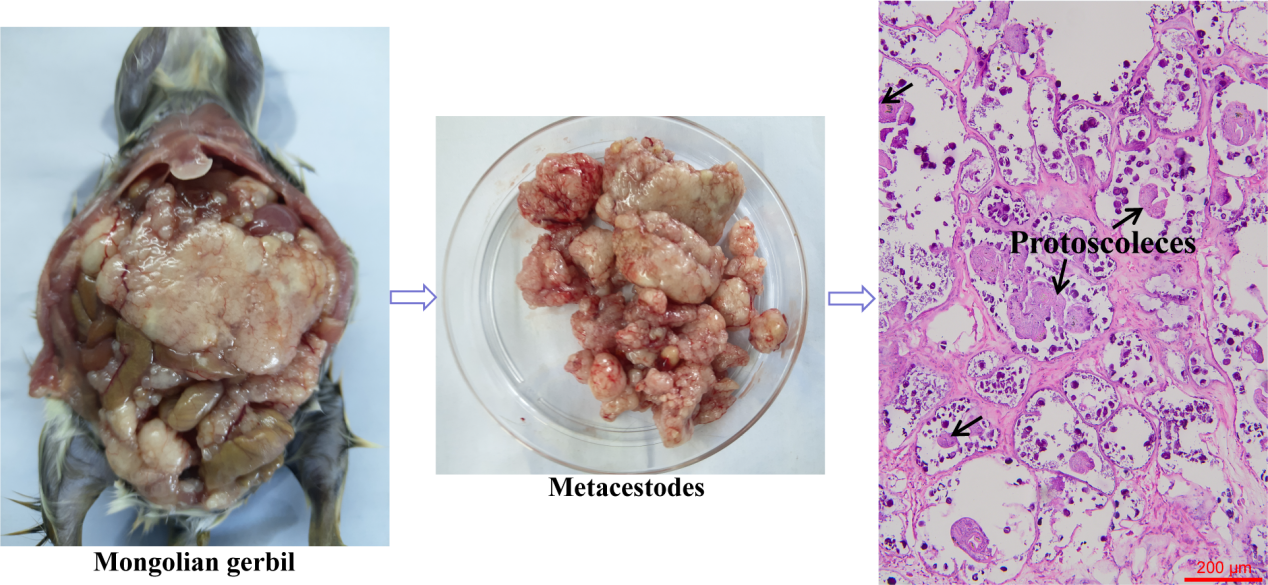


## **Additional file 3: Figure S3.** Isolation of metacestodes from Mongolian gerbil. The black arrows show the protoscoleces.
