# Supplementary material for: In vitro and in vivo efficacy of thiacloprid against Echinococcus multilocularis
Source: Parasit Vectors. 2021 Sep 6;14:450. doi: 10.1186/s13071-021-04952-7 (PMC8419995; doi:10.1186/s13071-021-04952-7)
Supplement: Supplementary file 4 — Additional file 4: Table S1. Specific primer sequences. [file 13071_2021_4952_MOESM4_ESM.doc]

**Additional file 4: Table S1. Specific primer sequences**

| **Genes** | **Sequences (5′ ‐ 3′)** |
| --- | --- |
| Collagen I | Forward: CTGGTGCTGAAGGCTCCCCT  Reverse: ATCGCCATTCTTGCCAGCGG |
| Collagen Ⅲ | Forward: GCTCCTGGAGGCAAGGGTGA  Reverse: AGAAGCACCAGGAGGGCCAG |
| MMP1 | Forward: ACACGCCAGATTTGCCAAGAGC  Reverse: GGAGAGTTGT CCCGATGATCTCCCC |
| MMP3 | Forward: TCTGGGAGGAGGTGACCCCA  Reverse: GCCAAGACTGTTCCAGGCCC |
| MMP9 | Forward: AGCCCCTGCTCCTGGCTCTC  Reverse: CTGCCAGCTGGGTGTCCGTG |
| MMP13 | Forward: ACTCCCTGTTGGTCCCTGCC  Reverse: TCCCGCAAGAGTCGCAGGAT |
| RPS18 | Forward: GCCAGGTTCTGGCCAACGGT  Reverse: CCCTGCGGCCAGTGGTCTTG |
