# Supplementary material for: In vitro and in vivo efficacy of thiacloprid against Echinococcus multilocularis
Source: Parasit Vectors. 2021 Sep 6;14:450. doi: 10.1186/s13071-021-04952-7 (PMC8419995; doi:10.1186/s13071-021-04952-7)
Supplement: Supplementary file 5 — Additional file 5: Table S2. Antibody information. [file 13071_2021_4952_MOESM5_ESM.docx]

| Antibodies | Source | Dilution ratio | Observed MW | Identifier |
| --- | --- | --- | --- | --- |
| COL1 | Abclonal | 1:800 | 125 kDa | A5786 |
| COL3 | Abclonal | 1:800 | 170 kDa | A3795 |
| MMP1 | Abclonal | 1:1000 | 54 kDa | A1191 |
| MMP3 | Abclonal | 1:1000 | 54 kDa | A1202 |
| MMP9 | Abclonal | 1:1000 | 85 kDa | A11402 |
| MMP13 | Abclonal | 1:1000 | 72 kDa | A11755 |
| β-actin | Abclonal | 1:1000 | 43 kDa | AC026 |

**Additional file 5: Table S2. Antibody information**
