# Supplementary figures and images for: In vitro and in vivo efficacy of thiacloprid against Echinococcus multilocularis
Source: Parasit Vectors. 2021 Sep 6;14:450. doi: 10.1186/s13071-021-04952-7 (PMC8419995; doi:10.1186/s13071-021-04952-7)

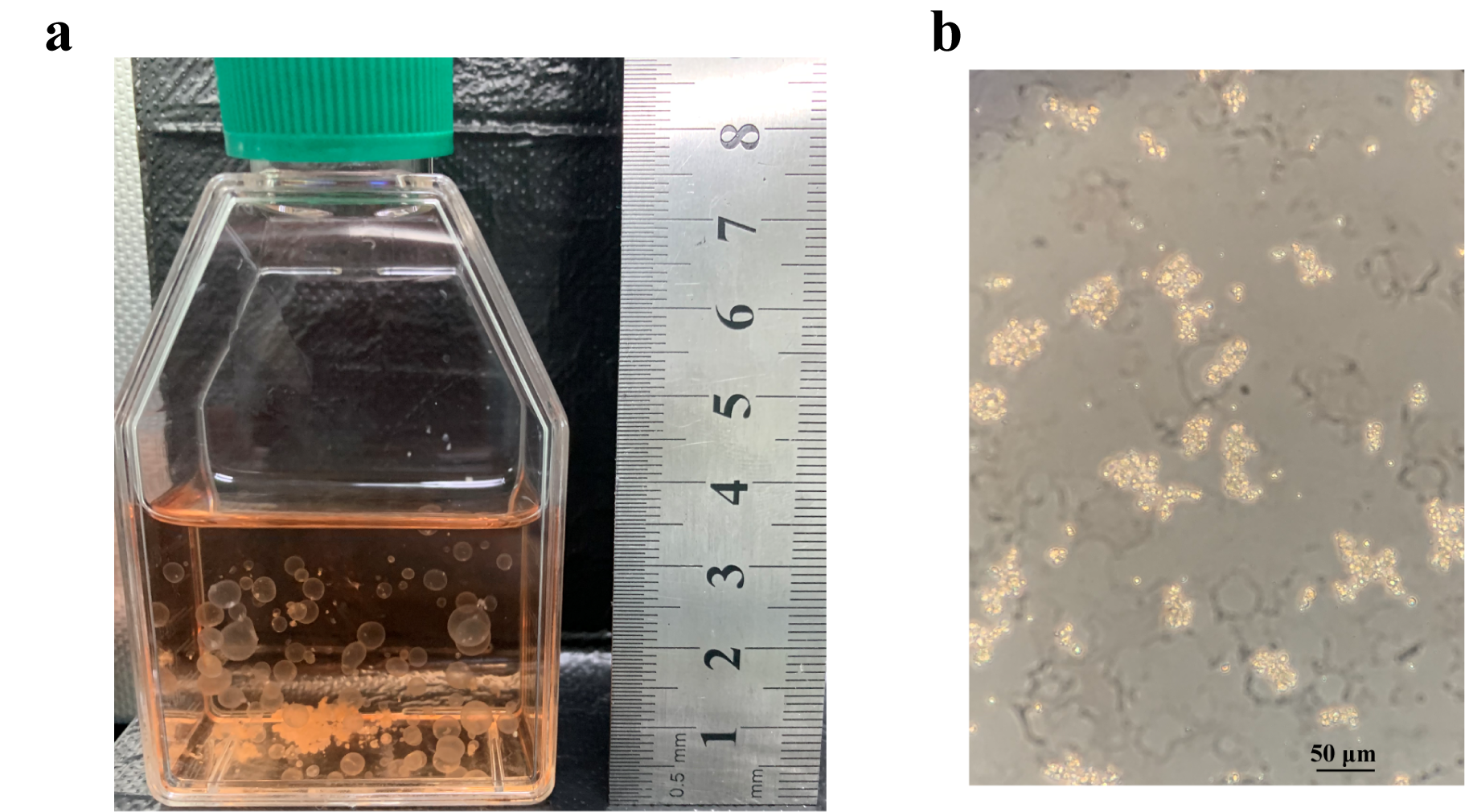


**Additional file 6: Figure S4. Metacestode vesicle (a) and germinal cells (b) cultured *in vitro.***

Supplement: Supplementary file 6 — Additional file 6: Figure S4. Metacestode vesicle (a) and germinal cells (b) cultured in vitro. [file 13071_2021_4952_MOESM6_ESM.doc]
