# Supplementary material for: In vitro and in vivo efficacy of thiacloprid against Echinococcus multilocularis
Source: Parasit Vectors. 2021 Sep 6;14:450. doi: 10.1186/s13071-021-04952-7 (PMC8419995; doi:10.1186/s13071-021-04952-7)
Supplement: Supplementary file 7 — Additional file 7: Table S3.Echinococcus multilocularis cholinesterase activity. [file 13071_2021_4952_MOESM7_ESM.docx]

**Additional file 7: Table S3. *E. multilocularis* Cholinesterase activity .**

| **Substrate** | **Inhibitor**  **(nm)** | **Cholinesterase activity (U/mg)** | | |
| --- | --- | --- | --- | --- |
|  |  | **Protoscoleces** | **Metacestodes** | **Germinal cells** |
| Acetylthiocholine odide | 0 | 11.02±0.59 | 5.37±0.89 | 6.37±0.75 |
|  | 4×10^4^ | 1.74±0.30** | 1.14±0.67** | 1.34±0.82** |
|  | 4×10^3^ | 2.35±0.20** | 1.89±0.63** | 1.79±0.37** |
|  | 4×10^2^ | 3.51±0.31** | 2.37±0.86** | 2.43±0.58** |
|  | 4×10^1^ | 4.22±0.33** | 3.08±0.94** | 3.17±0.61** |
|  | 4 | 4.70±0.36** | 3.86±0.73 | 4.12±0.83** |

U: one unit is 1 µmol of Substrate hydrolyzed per minute. The data were presented as the mean ± SD obtained from five independent experiments. ***P*<0.01 vs 0nm inhibitor.
