# Supplementary material for: In vitro and in vivo efficacy of thiacloprid against Echinococcus multilocularis
Source: Parasit Vectors. 2021 Sep 6;14:450. doi: 10.1186/s13071-021-04952-7 (PMC8419995; doi:10.1186/s13071-021-04952-7)
Supplement: Supplementary file 8 — Additional file 8: Figure S5. The cell viability of protoscoleces and germinal cells treated with thiacloprid for 1 h. a Survival of protoscoleces after treatment with thiacloprid. To evaluate the survival of protoscoleces, 0.1% eosin staining exclusion method was used. b Mitochondrial probe labeling of living cells. [file 13071_2021_4952_MOESM8_ESM.docx]

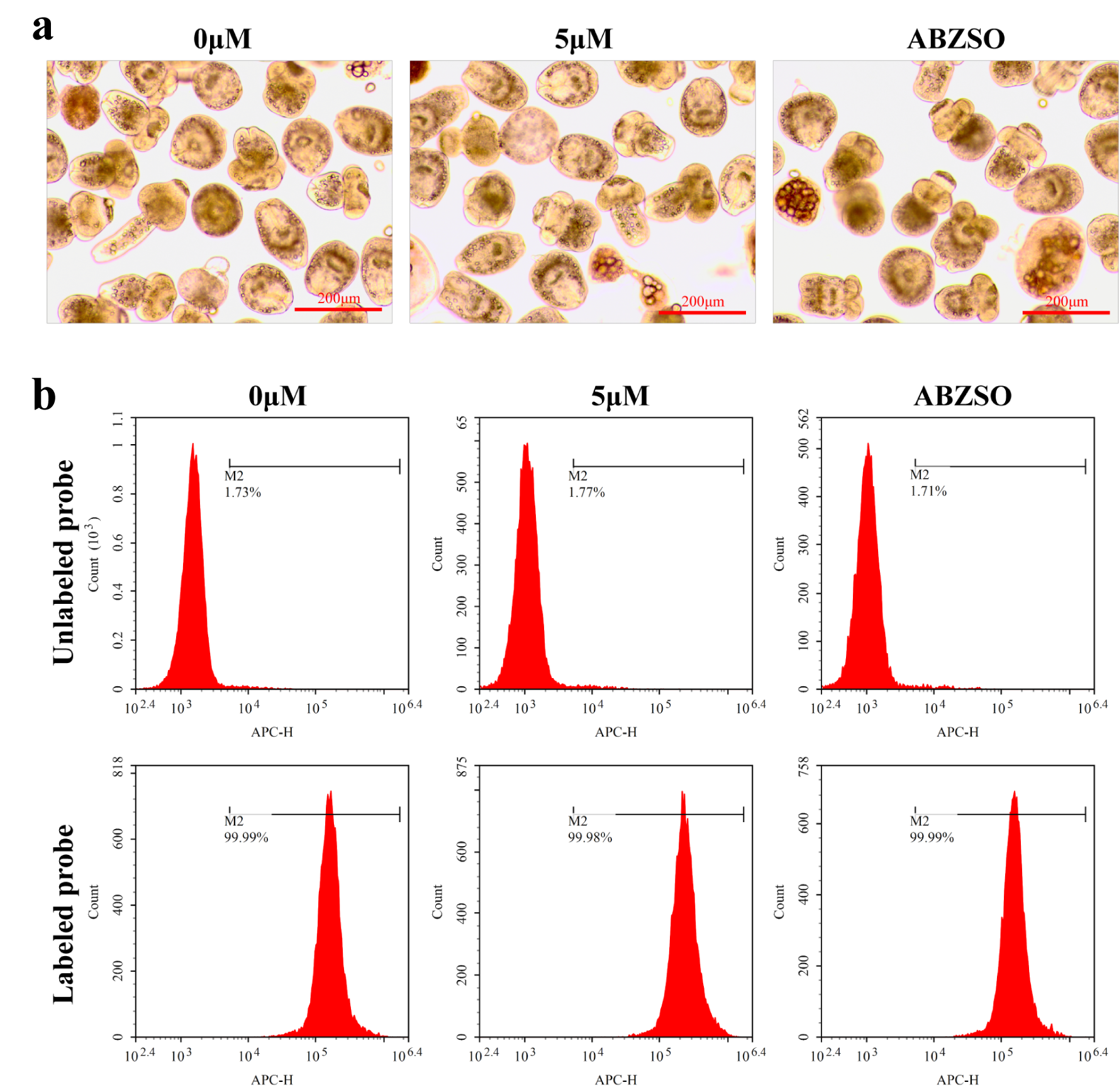


**Additional file 8: Figure S5. The cell viability of protoscoleces and germinal cells treated with thiacloprid for 1h.** **a** Survival of protoscoleces after treatment with thiacloprid. To evaluate the survival of protoscoleces, 0.1% eosin staining exclusion method was used. **b** Mitochondrial probe labeling of living cells.
