# Supplementary material for: In vitro and in vivo efficacy of thiacloprid against Echinococcus multilocularis
Source: Parasit Vectors. 2021 Sep 6;14:450. doi: 10.1186/s13071-021-04952-7 (PMC8419995; doi:10.1186/s13071-021-04952-7)
Supplement: Supplementary file 9 — Additional file 9: Figure S6. Metacestode in the abdominal cavity of mice. The black arrows showed metacestodes. [file 13071_2021_4952_MOESM9_ESM.docx]

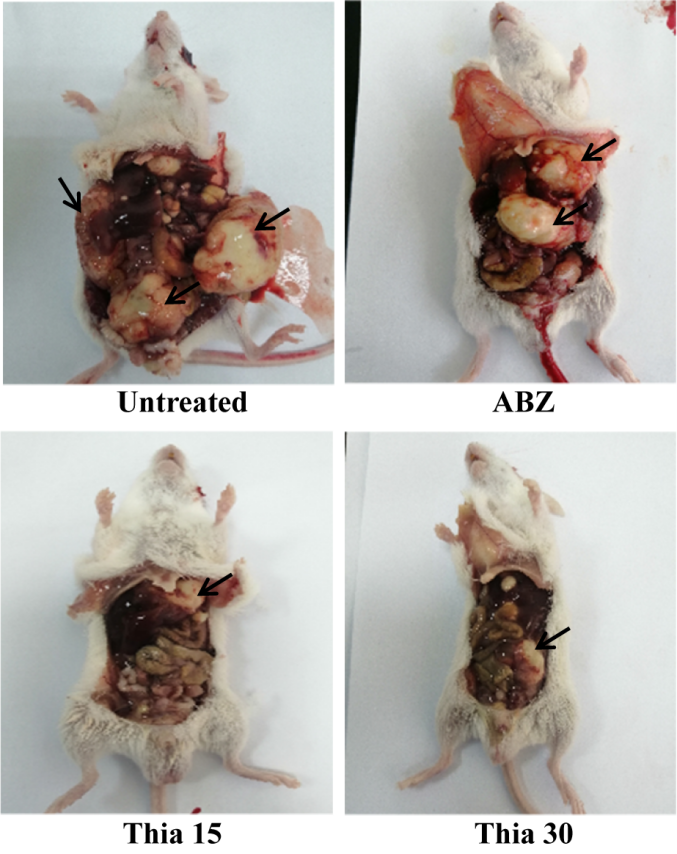


**Additional file 9: Figure S6. Metacestode in the abdominal cavity of mice.** The black arrows showed metacestodes.
