# Supplementary material for: In vitro and in vivo efficacy of thiacloprid against Echinococcus multilocularis
Source: Parasit Vectors. 2021 Sep 6;14:450. doi: 10.1186/s13071-021-04952-7 (PMC8419995; doi:10.1186/s13071-021-04952-7)
Supplement: Supplementary file 10 — Additional file 10: Figure S7. The center of the metacestodes showed caseous necrosis. HE staining showed caseous necrosis in the centre of the metacestode (scale bar = 400 μm). There is no structural granular and no residual shadow of the original tissue structure. [file 13071_2021_4952_MOESM10_ESM.docx]

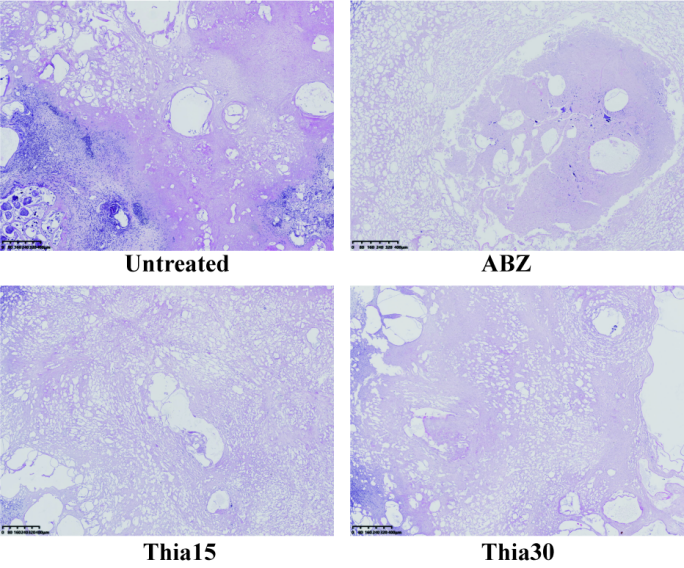


**Additional file 10: Figure S7.** **The centre of the metacestodes showed caseous necrosis.** HE staining showed caseous necrosis in the centre of the metacestode (scale bar=400 μm). There is no structural granular and no residual shadow of the original tissue structure.
