# Supplementary material for: In vitro and in vivo efficacy of thiacloprid against Echinococcus multilocularis
Source: Parasit Vectors. 2021 Sep 6;14:450. doi: 10.1186/s13071-021-04952-7 (PMC8419995; doi:10.1186/s13071-021-04952-7)
Supplement: Supplementary file 11 — Additional file 11: Table S4. Blood cell analysis of mice infected with E. multilocularis treated with thiacloprid (n = 6). [file 13071_2021_4952_MOESM11_ESM.docx]

**Additional file 11: Table S4. Blood cell analysis of mice infected with *E. multilocularis* treated with thiacloprid (n=6).**

| BALB/c mice | Control | Untreated | ABZ | Thia 15 | Thia 30 |
| --- | --- | --- | --- | --- | --- |
| WBC (109/L) | 13.38±1.26 | 30.60±4.95* | 27.84±4.27* | 25.10±3.86* | 27.60±3.10* |
| Eos (%) | 2.10±0.53 | 3.93±1.81 | 1.71±0.59 | 4.37±1.32^*^ | 2.45±0.71 |
| Neu (%) | 45.38±11.95 | 57.60±12.93 | 73.19±8.66* | 47.43±12.25 | 49.52±7.23 |
| Lym (%) | 41.58±12.11 | 21.55±7.15^*^ | 22.49±6.83^*^ | 41.27±10.25# | 50.38±15.14# |
| RBC (1012/L) | 10.36±0.81 | 8.18±2.32 | 9.60±1.12 | 9.22±0.60 | 8.71±1.50 |
| HGB (g/L) | 213.50±10.71 | 198.30±8.75 | 203.50±19.66 | 196.50±11.93 | 190.8±35.86 |

* *p*<0.05 vs Control; # *p*<0.05 vs Untreated.
