# Supplementary material for: In vitro and in vivo efficacy of thiacloprid against Echinococcus multilocularis
Source: Parasit Vectors. 2021 Sep 6;14:450. doi: 10.1186/s13071-021-04952-7 (PMC8419995; doi:10.1186/s13071-021-04952-7)
Supplement: Supplementary file 12 — Additional file 12: Figure S8. Immune cell infiltration in host tissue surrounding metacestode. After thiacloprid treatment, infiltration of neutrophils decreased (a) and infiltration of plasma cells, lymphocytes and macrophages increased in host tissue surrounding metacestode (b). The red arrow shows the neutrophils, the black arrow shows the lymphocytes, the green arrow shows the eosinophils, the blue arrow shows the plasma cells, and the yellow arrow shows the macrophages. [file 13071_2021_4952_MOESM12_ESM.docx]

**
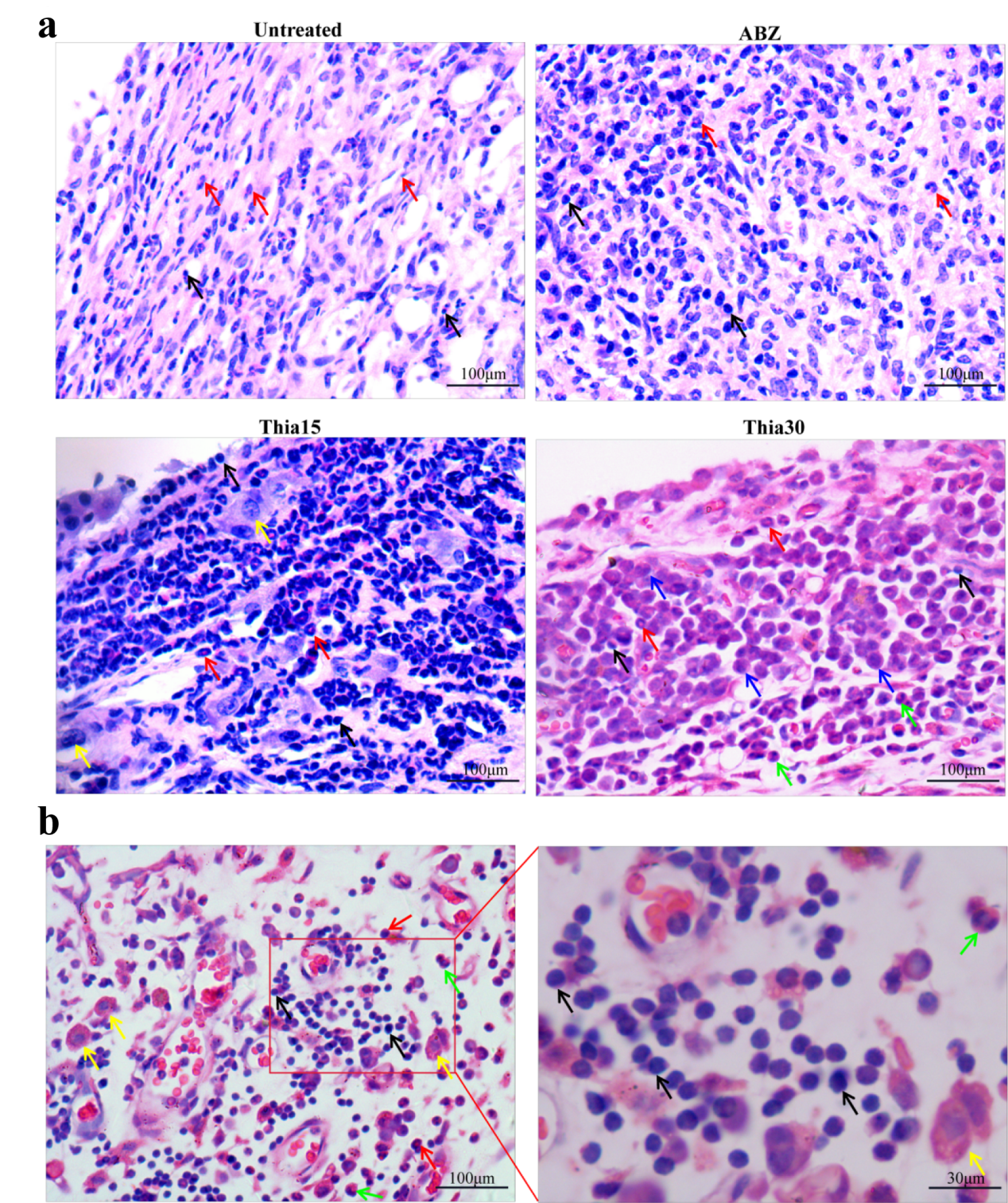
**

**Additional file 12: Figure S8. Immune cell infiltration in host tissue surrounding metacestode.** After thiacloprid treatment, infiltration of neutrophils decreased (**a**) and infiltration of plasma cells, lymphocytes and macrophages increased in host tissue surrounding metacestode (**b**). The red arrow shows the neutrophils, the black arrow shows the lymphocytes, the green arrow shows the eosinophils, the blue arrow shows the plasma cells, and the yellow arrow shows the macrophages.
